# Systemic inflammatory proteins in offspring following maternal probiotic supplementation for atopic dermatitis prevention

**DOI:** 10.1186/s12948-023-00186-3

**Published:** 2023-07-29

**Authors:** Dinastry Pramadita Zakiudin, Anne Dorthea Bjerkenes Rø, Vibeke Videm, Torbjørn Øien, Melanie Rae Simpson

**Affiliations:** 1 Department of Public Health and Nursing, NTNU, Norwegian University of Science and Technology, Håkon Jarls Gate 11, 7030 Trondheim, Norway; 2 Clinic for Laboratory Medicine, St Olavs Hospital, Trondheim, Norway; 3 Department of Clinical and Molecular Medicine, NTNU, Norwegian University of Science and Technology, Trondheim, Norway; 4 Department of Immunology and Transfusion Medicine, St. Olavs Hospital, Trondheim University Hospital, Trondheim, Norway

**Keywords:** Atopic dermatitis, Biomarkers, Children, Plasma, Probiotics

## Abstract

**Background:**

Maternal probiotic supplementation has a promising effect on atopic dermatitis (AD) prevention in infancy. In the randomised controlled study, Probiotics in the Prevention of Allergy among Children in Trondheim (ProPACT), maternal probiotics reduced the cumulative incidence of AD in their offspring by 40% at 2 years of age. However, our understanding on how probiotics prevented AD is still limited, and the role of inflammatory proteins in infants following maternal probiotic supplementation is unclear. We hypothesised that maternal probiotics lowered pro-inflammatory proteins and increased anti-inflammatory proteins in their 2-year-old children as a mechanism of AD prevention. We aimed to explore this hypothesis and the association between these proteins and the presence of AD, severity of AD, and the degree of preventive effect of probiotics.

**Methods:**

Plasma samples were collected from 2-year-old children (n = 202) during the ProPACT study, a randomised placebo-controlled trial of maternal probiotic supplementation. These samples were analysed for 92 inflammatory proteins using a multiplex proximity extension assay. Associations between inflammatory proteins and the presence and severity of AD, and the degree of preventive effect, was estimated individually using regression analysis and then collectively using unsupervised cluster analysis.

**Results:**

Several proteins were observed to differ between the groups. The probiotic group had lower CCL11 and IL-17C, while children with AD had higher IL-17C, MCP-4, uPA, and CD6. Cytokine CCL20 and IL-18 had moderate correlation (r = 0.35 and r = 0.46) with the severity of AD. The cluster analysis revealed that children in the cluster of samples with the highest value of immune checkpoint receptors and inflammatory suppressor enzymes showed the greatest AD preventive effect from probiotics.

**Conclusions:**

The proteins associated with both maternal probiotic supplementation and the presence and severity of AD warrant attention because of their potential biological relevance. Cluster analysis may provide a new insight when considering which subgroups benefit from probiotic supplementation. Larger studies are needed to confirm the results.

*Trial registration number:* The study was retrospectively registered at ClinicalTrials.gov (NCT00159523) on 12nd September 2005.

**Supplementary Information:**

The online version contains supplementary material available at 10.1186/s12948-023-00186-3.

## Background

Allergic diseases have been increasing in prevalence worldwide [1]. Environmental factors such as industrialisation, modern lifestyle, and pollutants may play an important role in this increase [1, 2]. These changes are thought to have led to reduced exposure of microbiota in early life [3], and several studies have linked microbiota disruptions in the first months of life to the development of allergic diseases [4, 5]. Even with their increased prevalence [1], we have limited knowledge about effective preventive strategies for allergic diseases [6, 7]. Atopic dermatitis (AD), or eczema, is the most common allergy-related disease in childhood [2] affecting up to 1 in 3 children by the age of 8 in Europe [8]. The burden of AD can be significant due to sleep disruptions and risk of skin infections and effective strategies for prevention is limited, yet important [9].

Several clinical trials have found that probiotic supplementation around the time of birth may prevent AD [10, 11] especially when using combinations of strains and regimes that include pre- and postnatal supplementation [12]. However, our understanding of how probiotics prevent AD in childhood is still limited [10, 13], and systemic biomarkers may provide insights into the mechanism. Although probiotics are reported to reduce pro-inflammatory and increase anti-inflammatory serum biomarkers in adults [14], no previous study has investigated the effect of maternal probiotic supplementation on the level of proteins associated with inflammation in their offspring [15].

In the randomised placebo-controlled study, Probiotics in the Prevention of Allergy among Children in Trondheim (ProPACT), short-term administration of probiotic bacteria given to a nonselected population of pregnant women reduced the cumulative incidence of AD in their offspring by 40% at 2 years old [11]. We hypothesised that maternal probiotics reduced pro-inflammatory proteins and increased anti-inflammatory proteins in their 2-year-old children as a mechanism of AD prevention. Our primary aim was to explore whether probiotics influenced plasma inflammatory proteins in children at 2 years of age. As secondary aims, we investigated if individual proteins were correlated with the presence and severity of AD, and if the degree of preventive effect was associated with different inflammatory protein profiles.

## Methods

### Participants and sample collection

The ProPACT study followed 415 pregnant women randomised to receive probiotics or placebo milk from 36 weeks of gestation until 3 months post-delivery while breastfeeding [16, 17]. The pregnant women were recruited from a nonselected population, and a computer-generated randomisation sequence allocated them to probiotic or placebo milk. The probiotic milk corresponded to a daily dose of 5 × 10^10^ colony-forming units (CFU) *Lactobacillus rhamnosus* GG (LGG), 5 × 10^10^ CFU *Bifidobacterium animalis subsp. lactis* Bb-12 (Bb-12) and 5 × 10^9^ CFU *Lactobacillus acidophilus* La-5 (La-5), whilst the placebo was fermented and pasteurised skim milk with similar taste and without probiotic bacteria. Information regarding demographics and risk factors for allergy-related diseases was obtained from questionnaires completed during pregnancy, and at the ages of 6 weeks, 1 year, and 2 years. A paediatrician examined all children at 2 years, and AD was defined using the U.K. working party’s diagnostic criteria for AD [18]. Additionally, children were encouraged to attend an examination by a trained nurse if they had an itchy rash for more than 4 weeks any time during the first year of life to ensure all cases were identified. The AD severity was assessed with the Nottingham Eczema Severity Score (NESS) [19].

Children who attended the clinical examination and with available samples at 2 years were eligible for inclusion in the current study. Ultimately, 202 children were included, 101 from the probiotic group and 101 from the placebo group. All participating mothers signed a written consent. The study was approved by the Regional Committee for Medical Research Ethics in Central Norway (097–03) and registered at ClinicalTrials.gov (NCT00159523).

### Plasma analyses

Heparin blood samples were collected from children at 2 years of age between December 2004 until April 2009 and diluted 1:1 with isotonic saline before plasma separation and storage at -80° C (further details in Additional file 1). Analysis of proteins were completed in October 2020.

Using the Olink® Target 96 Inflammation panel (Olink Proteomics, Uppsala, Sweden), 92 proteins associated with inflammation were analysed with a multiplex proximity extension (PEA) assay (complete list in Additional file 1: Table S1) [20]. The results were expressed as normalised protein expression (NPX) values which are arbitrary units on a Log2 scale, such that a one NPX unit increase corresponds to a doubling of the protein concentration for a given protein. As a relative quantification, the size of the NPX value can be compared within, but not between, proteins [21]. Different proteins with the same NPX value may still differ in their absolute concentrations.

### Statistical analyses

The statistical analyses were performed using Stata/IC 17 (StataCorp) and RStudio (version R 4.1.3). Descriptive variables were presented as mean (standard deviation (SD)) for continuous variables, and frequency (percentage) for categorical variables. Differences between groups in the detectability of proteins were analysed with Fisher’s exact test for categorical data to compare the proportion of samples with detectable expression of proteins. In analyses comparing protein expression we included 64 proteins which were above the limit of detection (LOD) in at least 50% of samples. For samples with NPX values below the LOD for these 64 proteins, we still used the provided NPX value in the quantitative analyses, as recommended by Olink [22]. To explore whether maternal probiotics supplementation influenced individual plasma protein expression we used the Linear Models for Microarray Data (*limma*) package [23]. The results are presented with fold change and p-values. Pairwise Pearson or Spearman’s rank correlation was done to find which protein(s) that correlated to AD severity score by NESS.

We used unsupervised hierarchical clustering to identify plasma inflammatory profiles in the offspring. This analysis strategy results in information about clustering of both samples and proteins by identifying which samples are similar across the measured proteins, as well as which proteins are correlated with each other across samples. To distinguish between the clustering results for inflammatory protein expression of plasma samples from the children and 92 proteins measured, we use the term “sample clusters” and “groups of proteins” throughout the results and discussion. The cluster analysis was performed using Ward linkage in combination with squared Euclidean distances between samples and correlation distance between proteins [24], and was visualised using a heatmap produced by pheatmap package in R software and displaying hierarchical trees for both samples and proteins (Additional file 1: Fig. S1). Post cluster analysis and visual inspection of the hierarchical trees indicated four clusters of samples and three groups of proteins (see Additional file 1). To investigate whether maternal probiotic supplementation affected the overall inflammatory protein profile, we examined the association between sample clustering and randomisation group using Fisher’s exact test. Likewise, the association between the sample clusters and cumulative AD was assessed using Fisher’s exact test. The risk ratio (RR) of AD for children in the probiotic versus placebo group was also calculated within each sample cluster to determine if the degree of preventive effect differed between the overall inflammatory protein profile.

The expression of individual proteins was compared between the four sample clusters using linear regression (Additional file 1: Tables S5–S7) and the results are presented graphically (Fig. 2).

## Results

**Fig. 1:**
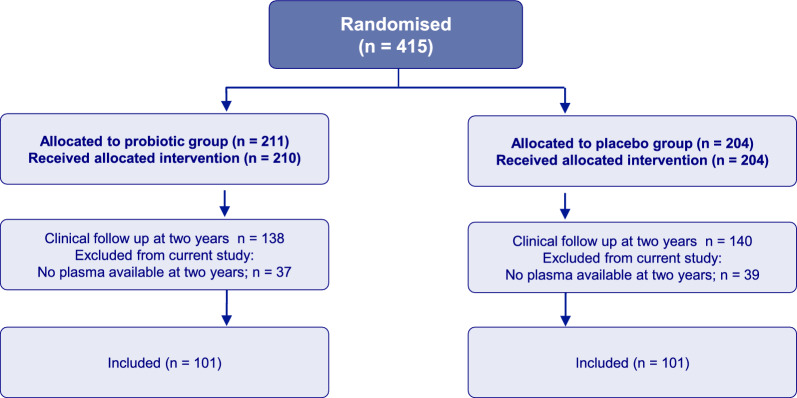
Flow of subjects in the probiotics and placebo groups

A total of 202 plasma samples were included (Fig. 1). The probiotic group had more males and more often had siblings than placebo group. Consistent with the overall results from the ProPACT study, the probiotic group in these analyses had a lower cumulative incidence and prevalence of current AD at 2 years of age, as well as a slightly lower proportion with current asthma (Table 1).

**Table 1:** Baseline characteristics of the study groups

Characteristics	Probiotic (n = 101)	%	Placebo (n = 101)	%	Incomplete data (%)
Male, gender	49	48	39	38	–
Born > 2 weeks before term (%)	11	12	7	7	17 (8)
Antibiotic use	39	39	41	42	5 (2)
Caesarean section delivery	9	12	11	15	55 (27)
Maternal smoking during pregnancy	3	3	3	3	4 (2)
Maternal atopy	51	50	53	53	2 (2)
Atopy in the family	66	65	70	69	1 (0.4)
Has siblings	46	45	36	36	–
Pets in the house	29	29	25	25	1 (0.4)
Birthweight(g) mean (SD)	3642 (476)	–	3589 (460)	-	–
Maternal age (years) mean (SD)	30.4 (3.7)	–	30.3 (4.1)	-	–
Atopy (IgE or skin prick test positive)	17	17	12	12	3 (1)
Cumulative Atopic Dermatitis	19	19	30	30	–
Current Atopic Dermatitis	7	7	14	14	–
Mild AD	7	7	11	11	–
Moderate AD	0	0	3	3	–
Asthma	4	4	9	9	–

*AD* atopic dermatitis. *SD* standard deviation. *IgE* immunoglobulin E. Cumulative Atopic Dermatitis – prevalence of atopic dermatitis in children since born until 2 years of age. Current Atopic Dermatitis – incidence of atopic dermatitis in children at 2 years of age clinical follow up

Among the 92 proteins analysed, 28 were found to be below the LOD in ≥ 50% samples. Nine proteins were not detected in any samples, 14 proteins were detected in less than 10% of samples, and five were detected in between 11 to 50% of samples (Additional file 1: Table S1). There was no substantial difference in the proportion of samples with detectable expression between the probiotic and placebo groups (Additional file 1: Table S1).

The 64 proteins considered for the subsequent statistical analyses are presented in the Additional file 1: Table S2. Most proteins had lower expression in the probiotic group, including CCL11 (eotaxin-1) and interleukin-17C (IL-17C) which had *p-*values < 0.05 (Table 2 and Additional file 1: Table S3). Similarly, IL-17C, monocyte chemoattractant protein-4 (MCP-4), urokinase-type plasminogen activator (uPA), and cluster of differentiation 6 (CD6) had higher expression in children with AD (Table 2 and Additional file 1: Table S4).

**Table 2:** *Limma* results for 10 proteins with lowest *p* values

Proteins (Top 10)	Average NPX (SD)		Fold Change	p-value
	Probiotics	Placebo		
IL-17C	1.18 (0.56)	1.49 (0.86)	0.807	**0.002**
CCL11	6.82 (0.54)	6.96 (0.47)	0.904	**0.04**
FGF19	5.71 (0.92)	5.96 (1.04)	0.838	0.06
MCP1	10.37 (0.41)	10.47 (0.43)	0.930	0.09
CXCL6	8.07 (0.55)	8.19 (0.47)	0.920	0.1
CDCP1	0.68 (0.34)	0.60 (0.29)	1.053	0.11
TNFRSF9	5.80 (0.48)	5.91 (0.44)	0.930	0.11
CCL23	6.52 (0.54)	6.63 (0.51)	0.922	0.11
CX3CL1	1.35 (0.45)	1.45 (0.56)	0.930	0.15
IL-8	4.23 (0.35)	4.31 (0.48)	0.942	0.15

Proteins (Top 10)	Average NPX (SD)		Fold Change	p-value
	AD (n = 49)	Without AD (n = 153)		
IL-17C	1.59 (0.92)	1.26 (0.65)	1.258	**0.005**
MCP4	13.55 (0.67)	13.29 (0.59)	1.195	**0.01**
uPA	8.32 (0.42)	8.17 (0.37)	1.113	**0.02**
CD6	5.64 (0.65)	5.45 (0.47)	1.144	**0.02**
CASP8	1.65 (0.70)	1.46 (0.54)	1.137	0.05
CST5	3.93 (0.84)	3.70 (0.72)	1.173	0.06
TNFSF14	3.58 (0.59)	3.44 (0.45)	1.103	0.08
SIRT2	3.30 (0.99)	3.00 (1.11)	1.227	0.09
IL-10RB	3.88 (0.52)	3.76 (4.40)	1.088	0.11
IL-8	4.35 (0.44)	4.25 (0. 41)	1.079	0.12

In bold: p-values < 0.05 were considered statistically significant. *NPX* normalised protein expression. *Limma* Linear Models for Microarray Data. IL –interleukin, *CCL* C–C motif chemokine ligand. *FGF* fibroblast growth factor, MCP – monocyte chemotactic protein, *CXCL* C-X-C motif chemokine ligand, Complement C1r/C1s, Uegf, Bmp1 (CUB) Domain Containing Protein. *TNFRSF* tumour necrosis factor receptor superfamily member. *uPA* urokinase-type plasminogen activator. *CD* cluster of differentiation. *CASP* caspase. *CST* cystatin-D. *TNFSF* tumour necrosis factor ligand superfamily member. *SIRT* sirtuin

*Limma* is a package for the analysis of gene expression microarray data, especially the use of linear models for analysing designed experiments and the assessment of differential expression. Empirical Bayesian methods are used to provide stable results even when the number of arrays is small. The linear model and differential expression functions apply to all gene expression technologies, including microarrays, RNA-seq and quantitative PCR

There was a moderate positive correlation between AD severity (NESS score) and IL-18 with r = 0.35 (*p* = 0.043, 95% CI 0.010 – 0.613) and CCL20 with r = 0.46 (*p* = 0.007, 95% CI 0.138 – 0.687).

Using hierarchical cluster analysis, we identified four clusters of samples with closest similarities according to Ward linkage and squared Euclidean distance (Table 3). We found no clear evidence that probiotic supplementation influenced the clustering of samples (p = 0.420). On the other hand, a statistically significant difference in cumulative incidence of AD between clusters was found, ranging from 15% in cluster 3 to 42% in cluster 4 (p = 0.027, Table 3). The observed preventive effect of maternal probiotics supplementation differed between clusters, with cluster 1 and cluster 3 having reduced RR of getting AD in the probiotic group (Table 3). We observed that cluster 1 had the highest proportion of pets in the house, maternal history of atopy, as well as the lowest proportion with siblings and allergic sensitisation at 2 years.

**Table 3:** Baseline characteristics of sample clusters defined by expression of inflammatory proteins in 2-year-old children

	Cluster 1 n = 56 (%)	Cluster 2 N = 59 (%)	Cluster 3 n = 75 (%)	Cluster 4 n = 12 (%)	*p* value (Fisher’s exact)
Probiotic	27 (48)	25 (42)	42 (56)	7 (58)	0.42
Male, gender	30 (54)	21 (36)	31 (41)	6 (50)	0.24
Atopy (IgE or skin prick positive)	5 (9)	12 (20)	9 (12)	3 (25)	0.17
Atopy in the family	40 (71)	39 (66)	48 (64)	9 (75)	0.84
Maternal atopy	32 (57)	28 (47)	39 (52)	5 (42)	0.61
Smoking in pregnancy	2 (3)	3 (5)	1 (1)	0	0.62
Has siblings	21 (37)	26 (44)	29 (39)	6 (50)	0.79
Pets in the house	22 (39)	11 (19)	19 (25)	2 (17)	0.08
Birthweight mean, g (SD)	3546 (485)	3635 (443)	3618 (464)	3650 (492)	0.75
Maternal age mean, (SD)	30.1, (3.9)	31.1, (3.8)	29.9, (4.0)	30.9, (3.6)	0.33
Cumulative AD	13 (23)	20 (34)	11 (15)	5 (42)	**0.03**
Current AD Severity					
Mild	6 (11)	8 (13)	3 (4)	1 (8)	0.78
Moderate	2 (2)	1 (2)	0 (0)	0 (0)	0.78
Relative risk of AD following maternal probiotic supplementation					
RR (95% CI)	0.26 (0.07–0.97)	1.09 (0.59–2.02)	0.61 (0.27–1.37)	1.87 (0.71–4.88)	–

The differences between groups were assessed the Fisher’s Exact test. In bold: P-values < 0.05 were considered statistically significant. *AD* atopic dermatitis. *SD* standard deviation. *IgE* immunoglobulin E. *RR* relative risk. *CI* confidence interval

**Fig. 2:**
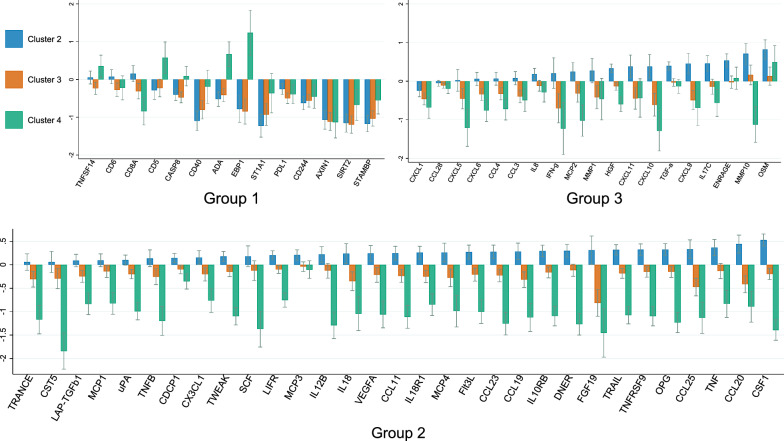
Protein expression level in the 3 groups of proteins with sample cluster 1 as baseline comparison

The hierarchical clustering also identified three groups of proteins which closely correlated to each other (Table 4 and Fig. 2). Overall, group 1 was characterised by T cell surface proteins with immune checkpoint receptor (ICR) function and enzymes with anti-inflammatory effect. These proteins had highest expression in sample cluster 1 which in turn had the lowest proportion of children with allergic sensitisation and the lowest relative risk of AD in the probiotic group. Cluster 1 especially had significantly high expression of axis inhibition protein 1 (AXIN1), CD244, programmed death-ligand 1 (PD-L1), signal-transducing adaptor molecule-binding protein (STAMBP), and sirtuin-2 (SIRT2) (Fig. 2). Both protein group 2 and 3 consisted of mostly chemokines, cytokine receptors, and growth factors which many are observed to play role in AD. Sample cluster 2 had higher expression of most of these proteins in group 2 and 3 while also having the highest proportions of current AD and maternal smoking. In contrast, the lowest expression of group 2 and 3 proteins were found in sample cluster 4 which clinically had the highest frequency of cumulative AD in the children and atopy both in children and the family.

**Table 4:** List of protein groups

Group 1 (n = 14)	Group 2 (n = 31)	Group 3 (n = 19)	Excluded proteins
Enzymes ADA, AXIN1, CASP8, SIRT2, ST1A1, STAMBP	Chemokines CCL11, CCL19, CCL20, CCL23, CCL25, CX3CL1, MCP1, MCP3, MCP4	Chemokines CCL3, CCL4, CCL28, CXCL1, CXCL5, CXCL6, CXCL9, CXCL10, CXCL11, MCP2	Interleukins IL-1A, IL-2, IL-4, IL-5, IL-6, IL-7, IL-10, IL-13, IL-17A, IL-20, IL-24, IL-33
T-cell surface CD244, CD5, CD6, CD8A, PD-L1	Growth factors CSF1, DNER, FGF19, (LAP)TGFB1, VEGFA	Growth factors HGF, TGFA	Cytokine receptors IL-10RA, 1L-15RA, IL-20RA, IL-22RA1, IL-2RB
Tumour necrosis factor ligand superfamily TNFSF14	Interleukins IL-12B, IL-18	Interleukins IL-17C, IL-8	Growth factors FGF5, FGF21, FGF23, NGFB, NTF3
Tumour necrosis factor and receptor superfamily CD40	Enzymes uPA, CST5	Enzymes MMP1, MMP10	Neurotrophic factor ARTN, GDNF, NRTN
Tumour suppressor 4E-BP1	Tumour necrosis factor and receptor superfamily OPG, TNF, TNFB, TNFRSF9, TRAIL, TRANCE, TWEAK	Growth regulator: IFNG, OSM	Cytokines LIF, TSLP
	Haematopoiesis CDCP1, FLT3LG, SCF	Antimicrobial peptide ENRAGE	SLAM family receptor SLAMF1
	Cytokine receptors IL-10RB, IL-18R1, LIFR		

*ADA* adenosine deaminase. *AXIN* axis inhibitor protein. *CASP* caspase- SIRT sirtuin. *ST* sulfotransferase. *STAMBP* signal-transducing adaptor molecule-binding protein. *CD* cluster of differentiation. *PD-L* programmed cell death. *TNFSF* tumour necrosis factor ligand superfamily member. 4E-BP eukaryotic translation initiation factor 4E-binding protein. *CCL* C–C motif chemokine ligand. *MCP* monocyte chemotactic protein. *CSF* macrophage colony-stimulating factor. DNER delta and notch-like epidermal growth factor-related receptor. *FGF* fibroblast growth factor. LAPTGFB1 latency-associated peptide transforming growth factor beta-1 proprotein. *VEGFA* vascular endothelial growth factor A. IL interleukin. *OPG* osteoprotegerin. *TNFRSF* tumour necrosis factor receptor superfamily member. *TRAIL* tumour necrosis factor-related apoptosis-inducing ligand. *TRANCE* tumour necrosis factor-related activation-induced cytokine, *TWEAK* tumour necrosis factor-related weak inducer of apoptosis. CDCP complement C1r/C1s, Uegf, Bmp1 (CUB) Domain Containing Protein. *FLT3LG* feline McDonough sarcoma (Fms)—related tyrosine kinase 3 ligand. *SCF* stem cell factor. *uPA* urokinase-type plasminogen activator. CST cystatin-D. *LIFR* leukaemia inhibitory factor receptor. *CXCL* C-X-C motif chemokine ligand. *HGF* hepatocyte growth factor. TGFA (pro)transforming growth factor alpha. *MMP* matrix metalloproteinase. IFNG interferon gamma. *OSM* oncostatin-M. ENRAGE extracellular newly identified receptor for advanced glycation end products binding protein. *FGF* fibroblast growth factor. *NGFB* nerve growth factor. NTF3 neurotrophin 3. *ARTN* artemin. *GDNF* glial cell-derived neurotrophic factor. *NRTN* neurturin. *TSLP* thymic stromal lymphopoietin. *SLAMF* signalling lymphocytic activation molecule family

## Discussion

In this study we have explored the role of multiple inflammatory proteins in the prevention of AD following maternal probiotic supplementation. We found that children in the probiotic group generally had lower expression of inflammatory proteins, especially CCL11 and IL-17C. The children with an AD diagnosis up to 2 years of age had higher expression of IL-17C, MCP-4, uPA, and CD6 whilst IL-18 and CCL20 may be moderately correlated with severity of AD in those children with AD. When considering the inflammatory protein profile collectively, we saw that the cluster of children with the greatest preventive effect following probiotic supplementation (sample cluster 1) also had higher expression of the protein group characterised by T cell surface proteins and enzymes with immunomodulatory functions (protein group 1).

Probiotics around the time of birth have been found to reduce the risk of AD both in the ProPACT study [11, 17] and meta-analyses [12], and the current study aimed to investigate if the preventive effect could be partially due to long term effects on the systemic inflammatory proteins of their offspring. Both CCL11 and IL-17C were observed to be lower in the probiotic group and they have previously been linked to AD, with the CCL11 playing an important role in AD severity [25]. The IL-17 cytokine family, which includes IL-17C, is produced by keratinocytes and is known to be overexpressed in AD skin lesions and serum [25–27]. While probiotics have been observed to reduce AD with suggested mechanism to be alterations of inflammatory markers in the breastmilk of the mothers [15], this is the first study to investigate the systemic inflammatory effect in offspring of mothers taking probiotic supplementation during pregnancy and while breastfeeding. A study in murine model observed that maternal probiotics suppressed the offspring’s IL-6, keratinocyte-derived cytokine (KC, also known as CXCL1), MCP-1, and IL-1β [28]. A small non-randomised and non-placebo-controlled study of 12-year-olds receiving probiotics did not find reduced plasma CCL11 or IL-17A (another member of the IL-17 family), yet reported reduced concentration levels of IL-12p40, IL-13, IL-15, IL-18, CCL2, and CCL24 [29]. Although these studies supported that probiotics may have an anti-inflammatory effect in the offspring, the changes were not observed in the same inflammatory biomarkers to those measured in our study and further research is needed.

In terms of associations between individual inflammatory proteins and the presence and severity of AD, we observed that higher expression of IL-17C, MCP-4, uPA and CD6 were seen in children with AD, and IL-18 and CCL20 were positively correlated with severity. Among those proteins in our study which associated with the presence of AD, IL-17C is expressed in AD lesions as described above, while MCP-4 is an eosinophil-specific chemotactic factor related to AD [30], uPA activity is linked to transepidermal water loss in dry AD skin [31] and CD6 is a T cell surface protein found to be overexpressed in AD skin [32]. Higher expression of both the IL-17 family cytokines and CD6 have previously been reported in adults with AD [25, 32], and MCP-4 has been associated with AD severity [30]. The correlation between severity and IL-18 and CCL20 is also consistent with other studies, with both observed to be related to severity in adults [33, 34] and IL-18 in older children [35]. Our findings suggest that this association may also be present in 2-year-old children, although another small study found no clear correlation between CCL20 and AD severity in 4-month-old infants [36].

Moving beyond the assessment of individual proteins for our primary aim, we also used hierarchical cluster analysis to examine if the overall inflammatory protein profile was associated with probiotic supplementation, the degree of preventive effect, or the presence of AD. The preventive effect of probiotic supplementation appeared to be greatest in sample cluster 1, which also included the children with a comparatively high proportion with family and maternal history of atopy. This is somewhat contradictory with the previous ProPACT study where the preventive effect was primarily seen among children without a family history of atopy [11]. Cluster 1 had the highest expression of protein group 1 which was characterised by high expression of ICRs such as PD-L1 and CD244, and inflammatory suppressor enzymes*.* ICRs trigger immunosuppressive signalling by suppressing autoreactive cells, which prevents excessive inflammation [37]. No previous studies have specifically discussed the roles of ICRs in AD in children, but it was observed that deficiencies in these proteins resulted in increased inflammation in mild AD in murine models [38]. Similarly, the AXIN1 enzyme has been associated with barrier dysfunction and higher AXIN1 concentration levels associated with lower AD risk [39]. The enzymes SIRT2 and STAMBP, which were also highest in sample cluster 1, suppress inflammation and have anti-inflammatory effects [40, 41]. Although sample cluster 1 had neither the highest expression of all anti-inflammatory nor the lowest expression of pro-inflammatory proteins, the presence and severity of AD is likely due to a balance between pro- and anti-inflammatory proteins [42], and it is difficult to separate the nuances of these relationships from our data. Furthermore, it is not possible to determine if the greater preventive effect seen in the probiotic group in cluster 1 represents an underlying inflammatory protein profile, alterations in the protein profile in early infancy, or if the inflammatory protein profile is a result of the AD prevention.

While the children in sample cluster 1 showed the greatest preventive effect of AD from probiotics, those in cluster 3 had the lowest prevalence of AD. This cluster consistently showed low expression of most of the measured proteins, especially tumour necrosis factor ligand superfamily member 14 (TNFSF14) which has previously been found to correlate with AD severity in adults [43]. On the other hand, the children in sample cluster 2 and 4 had higher risks of being diagnosed with AD, although with contrasting expression of inflammatory protein profiles. Overall, samples in cluster 2 had the highest expression of protein groups 2 and 3, and those in cluster 4 had the lowest expression. However, because there were very few samples in cluster 4, the results were interpreted with caution. Cluster 2 had particularly high expression of group 2 proteins which mostly consisted of pro-inflammatory cytokines found in AD [30].

The key strengths of this study are the double-blinded randomised design for probiotics intervention and novel use of the modern proteomics technologies and statistical analysis strategies to examine a wide range of inflammatory proteins in 2-year-old children. This study provides insight into the systemic inflammatory protein profile of 2-year-old children after maternal probiotic supplementation. Blood protein profiles after maternal probiotic supplementation in young children are scarce [14, 15, 44]. Using detailed clinical information based on validated diagnostic methods, we were able to consider the inflammatory protein profile in relation to the presence and severity of AD. Lastly, this study used hierarchical clustering which is a suitable and useful method for classifying large data with similarities [45]. This enabled us to compare protein profiles and the degree of preventative effect following probiotic supplementation.

Potential limitations of this study include the low level of detection of some proteins, the use of panel with a fixed set of proteins, and the relatively small samples size compared to the number of proteins analysed. Whilst the panel used in our study included several proteins previously related to AD and or type 2 inflammation, some of these could not be included in the analyses because of a low level of detection in a large number of samples. These included IL-4 and TSLP were detected in 1% and 3%, respectively, despite previous serum studies in young children reporting absolute concentrations above the LOD for the highly sensitive PEA technology used in the current study [25, 46]. Similarly, IL-5 and IL-13 were detected in only 4% and 3% of samples, respectively, and no samples had detectable levels of IL-33. The low levels of these proteins in our study may be due to sample dilution, the type of sample used (plasma), the children’s age [47], mild severity for most children with AD [48], and storage duration which might influence the concentrations found for some protein [49]. We note in particular, that some proteins which were reported as detectable in other studies of AD and healthy children but the proteins under LOD in our study also have been measured in peripheral blood mononuclear cells (PBMCs) [50] or serum [25, 51, 52] rather than plasma, or in plasma samples from older children [53–55]. In addition to the proteins below LOD, potentially interesting proteins were not available on the commercial panel, including some AD-related proteins (CCL17, CCL18, CCL22, and CCL27) and others associated with type 2 inflammation (IL-3, IL-9, IL-25, IL-31, and the eotaxins, CCL24 and CCL26) [56]. We cannot rule out that we would have found more differences or different patterns if these additional proteins could have been included, or if alternative collection, storage, and analysis methods allowed for quantification of the proteins found to have low levels of detection. Another limitation of this study is the comparison of multiple inflammatory markers in a relatively small number of participants in both the probiotics and placebo group. Due to the exploratory nature of the study, no statistical adjustment was performed for these multiple comparisons. The results should therefore be interpreted cautiously and still need to be confirmed in larger studies. Finally, we have no information about consumption of probiotic-containing food by mothers or infants between 3 months and 2 years of age when the samples were collected, however we have no reason to believe this would be different between the trial arms.

## Conclusion

In this randomised placebo-controlled exploratory study, maternal perinatal probiotic supplementation resulted in mostly lower expression of the measured plasma inflammatory biomarkers in the offspring at 2 years of age, especially CCL11 and IL-17C, while IL-17C, MCP-4, uPA and CD6 correlated with the presence and CCL20 and IL-18 with the severity of AD. Additionally, children with the greatest preventive effect of probiotics had the highest expression of the group of proteins characterised by T cell surface proteins with ICR function and suppressor enzyme of inflammation. Cluster analysis may provide a new insight when considering which subgroups benefit from probiotic supplementation. Further larger studies are needed to confirm the results.

## Supplementary Information

**Additional file 1.** Additional documentation. **Table S1.** Percentage of detectable Normalised Protein eXpression (NPX) levels of proteins. **Table S2.** Descriptive statistics of blood plasma proteins included in the statistical analyses. **Table S3.** *Limma *results for Probiotics and Placebo. **Table S4.** *Limma *results for Cumulative AD and Non-AD in children. **Table S5**. Expressions of proteins in first group by sample clusters. **Table S6.** Expressions of proteins in second group by sample clusters. **Table S7.** Expressions of proteins in third group by sample clusters. **Figure S1. **Inflammatory proteins of 2-year-old children following maternal probiotics suplementation.

## Data Availability

Primary patient data can be shared upon reasonable request to the corresponding author.

## Abbreviations

AD: Atopic Dermatitis
AXIN1: Axis inhibitor protein 1
CCL: C–C motif chemokine ligand
CD: Cluster of differentiation
CFU: Colony-forming units
CI: Confidence interval
CXCL: C-X-C motif chemokine ligand
ICRs: Immune checkpoint receptors
IL: Interleukin
LGG: *Lactobacillus* *rhamnosus* *GG*
*Limma*: Linear Models for Microarray Data
LOD: Limit of detection
NESS: Nottingham Eczema Severity Score
NPX: Normalised protein expression
PBMCs: Peripheral blood mononuclear cells
PD-L1: Programmed cell death ligand 1
PEA: Proximity extension assay
ProPACT: Probiotics in the Prevention of Allergy among Children in Trondheim
RR: Relative risk
SD: Standard deviation
SIRT2: NAD-dependent protein deacetylase sirtuin-2
STAMBP: Signal-transducing adaptor molecule-binding protein
TSLP: Thymic stromal lymphopoietin
uPA: Urokinase-type plasminogen activator
