# Supplementary material for: Systemic inflammatory proteins in offspring following maternal probiotic supplementation for atopic dermatitis prevention
Source: Clin Mol Allergy. 2023 Jul 29;21:5. doi: 10.1186/s12948-023-00186-3 (PMC10386175; doi:10.1186/s12948-023-00186-3)
Supplement: Supplementary file 1 — Additional file 1. Additional documentation. Table S1. Percentage of detectable Normalised Protein eXpression (NPX) levels of proteins. Table S2. Descriptive statistics of blood plasma proteins included in the statistical analyses. Table S3. Limma results for Probiotics and Placebo. Table S4. Limma results for Cumulative AD and Non-AD in children. Table S5. Expressions of proteins in first group by sample clusters. Table S6. Expressions of proteins in second group by sample clusters. Table S7. Expressions of proteins in third group by sample clusters. Figure S1. Inflammatory proteins of 2-year-old children following maternal probiotics suplementation. [file 12948_2023_186_MOESM1_ESM.docx]

**Additional file 1**

**Plasma preparation**

Heparin blood samples were collected from the children at 2 years of age. These samples were used for both isolation of mononuclear cells and plasma using density gradient centrifugation. Briefly, blood samples were diluted 1:1 with isotonic saline solution, then layered carefully over Lymphoprep (Fresenius Kabi, Oslo, Norway) and centrifuged. The plasma samples were collected from the top layer, and the mononuclear cells were collected from the cellular interface. The diluted plasma samples were stored at–80 °C until analysis.

**Post cluster analysis**

We use Calinski and Harabasz’s variance ratio criterion (VRC) or Calinski-Harabasz pseudo-F in Stata to determine the number of clusters in hierarchical clustering methods. The VRC with *n* objects and *k* clusters is defined as:

*VRC_k_ =* (*SS_B_* / (*k* – 1)) / (*SS_w_* / (n – *k*))

*SS_B_*= sum of the squares between the clusters

*SS_W_* = sum of the squares within the clusters

To determine the appropriate number of clusters, we chose the number that maximises the VRC. Because VRC usually decreases with a greater number of clusters, we computed the difference in the VRC values ωk of each cluster solution, using the following formula:

*wk* = (*VRC_k+1_* – *VRC_k_*) – (*VRC_k_* – *VRC_k-1_*)

The number of clusters *k* that minimise the value in *wk* indicates the best cluster solution.

Stata command: cluster stop wards linkage, rule(calinski) groups(2/11)

Stata Output:

| Number of clusters | Calinski/Harabasz pseudo-F |
| --- | --- |
| 2 | 30.16 |
| 3 | 25.68 |
| 4 | 25.14 |
| 5 | 22.36 |
| 6 | 20.66 |
| 7 | 19.12 |
| 8 | 17.72 |
| 9 | 16.63 |
| 10 | 15.79 |
| 11 | 14.86 |

Stata command: chomega

Output:

omega_3 is 3.942

omega_4 is -2.236

omega_5 is 1.074

omega_6 is 0.161

omega_7 is 0.144

omega_8 is 0.306

omega_9 is 0.257

omega_10 is -0.101

Minimum value of omega: -2.236 at 4 clusters. By using VRC method in Stata, the number of clusters that minimises value of omega resulted as 4 clusters of samples.

**Additional file 1: Table S1. Percentage of detectable Normalised Protein eXpression (NPX) levels of proteins**

| No | Proteins (Gene) /  Uniprot ID | Detectable NPX in all samples (%) | Detectable NPX level Probiotic group (%) | Detectable NPX level Placebo group (%) | *P* value  (Fisher’s exact) |
| --- | --- | --- | --- | --- | --- |
| Proteins included in the analysis (over 50% samples are above LOD): | | | | | |
| 1 | Adenosine Deaminase (ADA) / P00813 | 100 | 100 | 100 | N/A |
| 2 | Axis inhibitor protein 1 (AXIN1) / O15169 | 100 | 100 | 100 | N/A |
| 3 | C-C motif chemokine ligand 3 (CCL3) / P10147 | 100 | 100 | 100 | N/A |
| 4 | C-C motif chemokine ligand 4 (CCL4) / P13236 | 100 | 100 | 100 | N/A |
| 5 | C-C motif chemokine ligand 11 (CCL11, Eotaxin) / P51671 | 100 | 100 | 100 | N/A |
| 6 | C-C motif chemokine ligand 19 (CCL19) / Q99731 | 100 | 100 | 100 | N/A |
| 7 | C-C motif chemokine ligand 20 (CCL20) / P78556 | 100 | 100 | 100 | N/A |
| 8 | C-C motif chemokine ligand 23 (CCL23) / P55773 | 100 | 100 | 100 | N/A |
| 9 | C-C motif chemokine ligand 25 (CCL25) / O15444 | 100 | 100 | 100 | N/A |
| 10 | T-cell surface glycoprotein cluster of differentiation 5 (CD5) / P06127 | 100 | 100 | 100 | N/A |
| 11 | T-cell differentiation antigen cluster of differentiation 6 (CD6) / P30203 | 100 | 100 | 100 | N/A |
| 12 | T-cell surface glycoprotein cluster of differentiation 8 alpha chain (CD8A) / P01732 | 100 | 100 | 100 | N/A |
| 13 | Natural killer cell receptor 2B4, Cluster of differentiation 244 (CD244) / Q9BZW8 | 100 | 100 | 100 | N/A |
| 14 | Tumour necrosis factor receptor superfamily member 5,  Cluster of differentiation 40 (CD40) / P25942 | 100 | 100 | 100 | N/A |
| 15 | Macrophage colony-stimulating factor 1 (CSF1) / P09603 | 100 | 100 | 100 | N/A |
| 16 | Cystatin-D (CST5) / P28325 | 100 | 100 | 100 | N/A |
| 17 | Growth-regulated alpha protein, C-X-C motif chemokine ligand 1 (CXCL1) / P09341 | 100 | 100 | 100 | N/A |
| 18 | C-X-C motif chemokine ligand 5 (CXCL5) / P42830 | 100 | 100 | 100 | N/A |
| 19 | C-X-C motif chemokine ligand 6 (CXCL6) / P80162 | 100 | 100 | 100 | N/A |
| 20 | C-X-C motif chemokine ligand 9 (CXCL9) / Q07325 | 100 | 100 | 100 | N/A |
| 21 | C-X-C motif chemokine ligand 10 (CXCL10) / P02778 | 100 | 100 | 100 | N/A |
| 22 | C-X-C motif chemokine ligand 11 (CXCL11) / O14625 | 100 | 100 | 100 | N/A |
| 23 | Delta and notch-like epidermal growth factor-related receptor (DNER) / Q8NFT8 | 100 | 100 | 100 | N/A |
| 24 | Eukaryotic translation initiation factor 4E-binding protein 1, 4E-BP1 (EIF4EBP1) / Q13541 | 100 | 100 | 100 | N/A |
| 25 | Extracellular newly identified receptor for advanced glycation end products binding protein, Protein S100-A12 (EN-RAGE) / P80511 | 100 | 100 | 100 | N/A |
| 26 | Fibroblast growth factor 19 (FGF19) / O95750 | 100 | 100 | 100 | N/A |
| 27 | Feline McDonough sarcoma (Fms) - related tyrosine kinase 3 ligand (FLT3LG) / P49771 | 100 | 100 | 100 | N/A |
| 28 | HGF (Hepatocyte growth factor) / P14210 | 100 | 100 | 100 | N/A |
| 29 | Interleukin-10 receptor subunit beta (IL-10RB) / Q08334 | 100 | 100 | 100 | N/A |
| 30 | Interleukin-12 subunit beta (IL-12B) / P29460 | 100 | 100 | 100 | N/A |
| 31 | Interleukin-18 (IL-18) / Q14116 | 100 | 100 | 100 | N/A |
| 32 | Interleukin-18 receptor 1 (IL-18R1) / Q13478 | 100 | 100 | 100 | N/A |
| 33 | Interleukin -8 (IL-8) / P10145 | 100 | 100 | 100 | N/A |
| 34 | (Latency-associated peptide) transforming growth factor beta-1 proprotein (LAPTGFB1, TGFB1) / P01137 | 100 | 100 | 100 | N/A |
| 35 | Monocyte chemotactic protein-1 (MCP-1) / P13500 | 100 | 100 | 100 | N/A |
| 36 | Monocyte chemotactic protein-2 (MCP-2) / P80075 | 100 | 100 | 100 | N/A |
| 37 | Monocyte chemotactic protein-4 (MCP-4) / Q99616 | 100 | 100 | 100 | N/A |
| 38 | Matrix metalloproteinase-1, Interstitial collagenase (MMP1) / P03956 | 100 | 100 | 100 | N/A |
| 39 | Matrix metalloproteinase-10, Stromelysin-2 (MMP10) / P09238 | 100 | 100 | 100 | N/A |
| 40 | Osteoprotegerin (OPG) / O00300 | 100 | 100 | 100 | N/A |
| 41 | Oncostatin-M (OSM) / P13725 | 100 | 100 | 100 | N/A |
| 42 | Programmed cell death ligand 1, PD-L1, (CD274) / Q9NZQ7 | 100 | 100 | 100 | N/A |
| 43 | Stem cell factor (SCF) / P21583 | 100 | 100 | 100 | N/A |
| 44 | Signal-tranducing adaptor molecule binding protein (STAMBP) / O95630 | 100 | 100 | 100 | N/A |
| 45 | (Pro)Transforming growth factor alpha (TGFA) / P01135 | 100 | 100 | 100 | N/A |
| 46 | Tumour necrosis factor (TNF) / P01375 | 100 | 100 | 100 | N/A |
| 47 | Tumour necrosis factor beta, Lymphotoxin-alpha (TNFB) / P01374 | 100 | 100 | 100 | N/A |
| 48 | Tumour necrosis factor receptor superfamily member 9 (TNFRSF9) / Q07011 | 100 | 100 | 100 | N/A |
| 49 | Tumour necrosis factor ligand superfamily member 14 (TNFSF14) / O43557 | 100 | 100 | 100 | N/A |
| 50 | Tumour necrosis factor-related apoptosis-inducing ligand (TRAIL) / P50591 | 100 | 100 | 100 | N/A |
| 51 | Tumour necrosis factor-related activation-induced cytokine (TRANCE) / O14788 | 100 | 100 | 100 | N/A |
| 52 | Tumour necrosis factor-related weak inducer of apoptosis (TWEAK) / O43508 | 100 | 100 | 100 | N/A |
| 53 | Urokinase-type plasminogen activator, uPA (PLAU) / P00749 | 100 | 100 | 100 | N/A |
| 54 | Vascular endothelial growth factor A (VEGFA) / P15692 | 100 | 100 | 100 | N/A |
| 55 | Interferon gamma (IFNG) / P01579 | 100 | 100 | 99 | 1.000 |
| 56 | Sulfotransferase 1A1 (ST1A1)/P50225 | 97 | 99 | 100 | 1.000 |
| 57 | Leukaemia inhibitory factor receptor (LIFR) / P42702 | 98 | 97 | 98 | 0.999 |
| 58 | C-X3-C motif ligand 1, Fractalkine (CX3CL1) / P78423 | 85 | 83 | 86 | 0.697 |
| 59 | Caspase-8 (CASP-8) / Q14790 | 77 | 78 | 80 | 0.862 |
| 60 | NAD-dependent protein deacetylase sirtuin-2 (SIRT2) / Q8IXJ6 | 73 | 78 | 79 | 0.999 |
| 61 | Monocyte chemotactic protein-3 (MCP3) / P80098 | 62 | 59 | 65 | 0.468 |
| 62 | C-C motif chemokine ligand 28 (CCL28)/ Q9NRJ3 | 61 | 50 | 56 | 0.474 |
| 63 | Interleukin-17C, IL-17C (IL17C) / Q9P0M4 | 57 | 51 | 62 | 0.118 |
| 64 | Complement C1r/C1s, Uegf, Bmp1 (CUB) Domain Containing Protein 1 (CDCP1) / Q9H5V8 | 56 | 58 | 50 | 0.259 |
| Proteins excluded in the analysis (less than 50% samples are above LOD) | | | | | |
| 1 | Interleukin-7, IL-7 (IL7) / P13232 | 39 | 39 | 39 | 0.999 |
| 2 | Interleukin-10, IL-10 (IL10) / P22301 | 37 | 33 | 40 | 0.307 |
| 3 | Interleukin-17A, IL-17A (IL17A) / Q16552 | 31 | 27 | 25 | 0.872 |
| 4 | Fibroblast growth factor 21 (FGF21) / Q9NSA1 | 19 | 16 | 14 | 0.843 |
| 5 | Interleukin-6, IL-6 (IL6) / P05231 | 13 | 11 | 15 | 0.529 |
| 6 | Interleukin-10 receptor subunit alpha, IL-10RA (IL10RA) / Q13651 | 7 | 9 | 6 | 0.593 |
| 7 | Neurotrophin-3 (NTF3) / P20783 | 9 | 9 | 6 | 0.407 |
| 8 | Thymic stromal lymphopoietin (TSLP) / Q969D9 | 3 | 3 | 3 | 0.999 |
| 9 | Artemin (ARTN) / Q5T4W7 | 2 | 3 | 2 | 0.999 |
| 10 | Interleukin-13, IL-13 (IL13) / P35225 | 3 | 3 | 2 | 0.999 |
| 11 | Interleukin-24, IL-24 (IL24) / Q13007 | 2 | 3 | 2 | 0.999 |
| 12 | Interleukin-15 receptor subunit alpha, IL-15RA (IL15RA) / Q13261 | 4 | 3 | 1 | 0.621 |
| 13 | Signaling lymphocytic activation molecule 1 (SLAMF1) / Q13291 | 4 | 1 | 3 | 0.621 |
| 14 | Fibroblast growth factor 5 (FGF5) / P12034 | 1 | 1 | 2 | 0.497 |
| 15 | Interleukin-4, IL-4 (IL4) / P05112 | 1 | 1 | 2 | 0.999 |
| 16 | Interleukin-1 alpha, IL-1A (IL1A) / P01583 | 1 | 3 | 0 | 0.246 |
| 17 | Interleukin-5, IL-5 (IL5) / P05113 | 4 | 0 | 1 | 1.000 |
| 18 | Interleukin-20, IL-20 (IL20) / Q9NYY1 | 1 | 0 | 1 | 1.000 |
| 19 | Neurturin (NRTN) / Q99748 | 1 | 0 | 1 | 1.000 |
| 20 | Beta-nerve growth factor (NGFB) / P01138 | 0 | 0 | 0 | N/A |
| 21 | Fibroblast growth factor 23 (FGF23) / Q9GZV9 | 0 | 0 | 0 | N/A |
| 22 | Glial cell line-derived neurotrophic factor (GDNF) / P39905 | 0 | 0 | 0 | N/A |
| 23 | Interleukin-2, IL-2 (IL2) / P60568 | 0 | 0 | 0 | N/A |
| 24 | Interleukin-2 receptor subunit beta, IL-2RB (IL2RB)/ P14784 | 0 | 0 | 0 | N/A |
| 25 | Interleukin-20 receptor subunit alpha, IL-20RA (IL20RA) / Q9UHF4 | 0 | 0 | 0 | N/A |
| 26 | Interleukin-22 receptor subunit alpha 1, IL-22RA1 (IL22RA1) / Q8N6P7 | 0 | 0 | 0 | N/A |
| 27 | Interleukin-33, IL-33 (IL33) / O95760 | 0 | 0 | 0 | N/A |
| 28 | Leukaemia inhibitory factor (LIF) / P15018 | 0 | 0 | 0 | N/A |

UniProt ID – Universal Protein identifier. NPX – normalised protein expression. LOD – limit of detection. N/A – not available.

**Additional file 1:Table S2.** Descriptive statistics of blood plasma proteins included in the statistical analyses

| Proteins | Total group | | Probiotic, mean NPX (SD) | Placebo,  mean NPX (SD) |
| --- | --- | --- | --- | --- |
|  | **Mean NPX (SD)** | **Median NPX (Range)** |  |  |
| Tumour suppressors | | | | |
| 4E-BP1 | 6.08 (1.1) | 5.83 (4.25 – 10.06) | 6.07 (1.12) | 6.10 (1.08) |
| T-cell surface | | | | |
| CD244 | 5.06 (0.55) | 5.03 (3.41 – 7.41) | 5.02 (0.53) | 5.10 (0.57) |
| CD5 | 6.33 (0.69) | 6.33 (4.71 – 9.68) | 6.31 (0.68) | 6.35 (0.71) |
| CD6 | 5.49 (0.53) | 5.48 (3.98 – 7.27) | 5.47 (0.49) | 5.53 (0.56) |
| CD8A | 7.84 (0.63) | 7.89 (5.63 – 10.71) | 7.82 (0.62) | 7.86 (0.65) |
| PD-L1 | 3.57 (0.44) | 3.54 (2.33 – 5.72) | 3.55 (0.41) | 3.59 (0.48) |
| Tumour necrosis factor and receptor superfamily | | | | |
| CD40 | 10.48 (0.82) | 10.44 (8.86 –13.53) | 10.40 (0.82) | 10.56 (0.82) |
| OPG | 7.61 (0.49) | 7.66 (5.37 – 8.81) | 7.56 (0.48) | 7.65 (0.50) |
| TNF | 1.75 (0.55) | 1.69 (0.52 – 5.48) | 1.73 (0.55) | 1.78 (0.55) |
| TNFB | 4.48 (0.58) | 4.49 (2.78 – 5.79) | 4.45 (0.61) | 4.51 (0.55) |
| TNFRSF9 | 5.85 (0.47) | 5.89 (4.22 – 6.89) | 5.80 (0.48) | 5.91 (0.44) |
| TRAIL | 6.02 (0.44) | 6.05 (4.70 – 7.21) | 6.00 (0.43) | 6.04 (0.46) |
| TRANCE | 4.05 (0.57) | 4.12 (2.43 -5.42) | 4.03 (0.54) | 4.06 (0.59) |
| TWEAK | 6.89 (0.41) | 6.94 (5.56 -7.71) | 6.86 (0.404) | 6.94 (0.42) |
| Tumour necrosis factor ligand superfamily | | | | |
| TNFSF14 | 3.48 (0.49) | 3.40 (2.50 – 6.36) | 3.46 (0.48) | 3. 49 (0.49) |
| Chemokines | | | | |
| CCL3 | 3.96 (0.51) | 3.94 (2.87 – 6.46) | 3.91 (0.49) | 4.01 (0.54) |
| CCL4 | 4.49 (0.50) | 4.49 (3.25 – 6.95) | 3. 91 (0.43) | 4.54 (0.56) |
| CCL11 | 6.89 (0.51) | 6.90 (4.76 – 8.22) | 6.82 (0.54) | 6.96 (0.472 |
| CCL19 | 6.64 (0.59) | 6.65 (4.98 – 9.14) | 6.64 (0.61) | 6.66 (0.58) |
| CCL20 | 6.22 (0.66) | 6.12 (4.70 – 8.52) | 6.16 (0.63) | 6.29 (0.68) |
| CCL23 | 6.58 (0.52) | 6.59 (4.79 -7.61) | 6.52 (0.54) | 6.63 (0.51) |
| CCL25 | 3.85 (0.67) | 3.82 (2.03 – 5.89) | 3.81 (0.65) | 3.89 (0.70) |
| CCL28 | 0.95 (0.37) | 1.09 (0.53 -2.19) | 0.96 (0.35) | 0.94 (0.39) |
| CX3CL1 | 1.39 (0.51) | 1.46 (0.53 – 4.41) | 1.35 (0.45) | 1.45 (0.56) |
| CXCL1 | 9.29 (0.49) | 9.27 (7.93 – 10.77) | 9.27 (0.44) | 9.31 (0.53) |
| CXCL5 | 10.55 (0.83) | 10.56 (7.83 – 2.83) | 10.49 (0.91) | 10.60 (0.73) |
| CXCL6 | 8.13 (0.51) | 8.15 (6.55 – 9.45) | 8.07 (0.55) | 8.19 (0.47) |
| CXCL9 | 5.13 (0.82) | 5.02 (3.43 – 8.41) | 5.14 (0.88) | 5.11 (0.77) |
| CXCL10 | 7.05 (0.96) | 6.86 (5.38 – 10.83) | 7.04 (0.94) | 7.07 (0.99) |
| CXCL11 | 9.29 (0.86) | 9.17 (7.46 -12.81) | 9.30 (0.80) | 9.27 (0.92) |
| MCP1 | 10.42 (0.43) | 10.41 (8.93 – 1.84) | 10.37 (0.41) | 10.47 (0.43) |
| MCP2 | 7.07 (0.71) | 7.02 (5.24 – 9.52) | 7.07 (0.72) | 7.06 (0.71) |
| MCP3 | 1.49 (0.56) | 1.73 (0.81 – 2.61) | 1.45 (0.56) | 1.54 (0.57) |
| MCP4 | 13.35 (0.62) | 13.32 (11.56-14.99) | 13.31 (0.63) | 13.39 (0.61) |
| Interleukins | | | | |
| IL-8 | 4.27 (0.42) | 4.24 (3.26 – 6.19) | 4.23 (0.35) | 4.31 (0.48) |
| IL-12B | 5.42 (0.56) | 5.45 (3.48 – 6.85) | 5.44 (0.56) | 5.40 (0.57) |
| IL-17C | 1.34 (0.74) | 1.41 (0.67 -5.72) | 1.18 (0.555) | 1.49 (0.86) |
| IL-18 | 7.18 (0.66) | 7.17(5.32 – 9.37) | 7.21 (0.73) | 7.15 (0.59) |
| Cytokine receptors | | | | |
| IL-10RB | 3.79 (0.46) | 3.81(2.25 – 4.89) | 3.76 (0.47) | 3.81 (0.46) |
| IL-18R1 | 5.97 (0.47) | 5.95 (4.62 – 7.44) | 5.94 (0.45) | 6.01 (0.48) |
| LIFR | 1.86 (0.36) | 1.90 (0.56 – 2.68) | 1.85 (0.36) | 1.87 (0.36) |
| Growth regulator | | | | |
| IFNG | 5.69 (1.18) | 5.49 (1.55 -10.84) | 5.73 (1.26) | 5.65 (1.10) |
| OSM | 3.24 (0.75) | 3.13 (1.58 – 5.85) | 3.23 (0.73) | 3.25 (0.78) |
| Enzymes | | | | |
| ADA | 4.87 (0.60) | 4.76 (4.01 -8.70) | 4.85 (0.58) | 4.89 (0.63) |
| AXIN1 | 3.80 (0.83) | 3.83 (1.37 – 6.15) | 3.75 (0.80) | 3.86 (0.86) |
| CASP8 | 1.50 (0.58) | 1.55 (0.61 – 4.38) | 1.46 (0.55) | 1.56 (0.62) |
| CST5 | 3.76 (0.75) | 3.79 (1.20 – 6.52) | 3.73 (0.76) | 3.79 (0.75) |
| MMP1 | 11.97 (0.90) | 11.97 (9.31 –14.44) | 12.00 (0.86) | 11.93 (0.94) |
| MMP10 | 7.66 (0.85) | 7.68 (5.29 – 9.68) | 7.67 (0.91) | 7.65 (0.79) |
| SIRT2 | 3.07(1.09) | 3.21 (1.28 – 6.32) | 3.04 (1.05) | 3.11 (1.13) |
| ST1A1 | 3.49 (0.96) | 3.67 (0.57 – 5.94) | 3.44 (0.94) | 3.56 (0.97) |
| STAMBP | 3.88 (0.75) | 3.89 (2.12 – 6.81) | 3.84 (0.71) | 3.92 (0.79) |
| uPA | 8.21 (0.38) | 8.23 (6.81 – 9.05) | 8.17 (0.38) | 8.24 (0.39) |
| Growth factors | | | | |
| CSF1 | 7.84 (0.57) | 7.84 (5.49 – 9.21) | 7.79 (0.59) | 7.88 (0.55) |
| DNER | 6.88 (0.51) | 6.93 (5.09 – 7.97) | 6.83 (0.49) | 6.93 (0.52) |
| FGF19 | 5.83 (0.99) | 5.90 (3.35 – 8.37) | 5.71 (0.92) | 5.96 (1.04) |
| HGF | 5.51 (0.38) | 5.51 (4.27 – 6.71) | 5.47 (0.38) | 5.54 (0.38) |
| LAPTGFB1 | 4.67 (0.43) | 4.72 (3.36 – 5.74) | 4.64 (0.404) | 4.71 (0.45) |
| TGFA | 1.55 (0.35) | 1.53 (0.79 – 3.09) | 1.54 (0.33) | 1.57 (0.36) |
| VEGFA | 8.79 (0.55) | 8.74 (7.50 – 10.54) | 8.78 (0.56) | 8.79 (0.54) |
| Haematopoiesis | | | | |
| CDCP1 | 0.64 (0.32) | 0.69 (0.33 – 1.92) | 0.68 (0.34) | 0.60 (0.29) |
| FLT3LG | 6.52 (0.50) | 6.55 (4.97 – 7.93) | 6.49 (0.48) | 6.55 (0.52) |
| SCF | 6.58 (0.69) | 6.68 (4.26 – 8.29) | 6.57 (0.73) | 6.59 (0.66) |
| Antimicrobial peptide | | | | |
| EN-RAGE | 2.09 (0.52) | 1.99 (1.13 – 3.82) | 2.06 (0.46) | 2.12 (0.57) |

NPX – normalised protein expression. SD- standard deviation. ADA – adenosine deaminase. AXIN – axis inhibitor protein, CASP – caspase. SIRT – sirtuin, ST – sulfotransferase. STAMBP – signal-transducing adaptor molecule-binding protein. CD – cluster of differentiation. PD-L – programmed cell death. TNFSF – tumour necrosis factor ligand superfamily member. 4E-BP – eukaryotic translation initiation factor 4E-binding protein. CCL – C-C motif chemokine ligand. MCP – monocyte chemotactic protein. CSF – macrophage colony-stimulating factor. DNER – delta and notch-like epidermal growth factor-related receptor. FGF – fibroblast growth factor. LAPTGFB1 – latency-associated peptide transforming growth factor beta-1 proprotein. VEGFA – vascular endothelial growth factor A. IL – interleukin. OPG – osteoprotegerin. TNFRSF – tumour necrosis factor receptor superfamily member. TRAIL – tumour necrosis factor-related apoptosis-inducing ligand. TRANCE – tumour necrosis factor-related activation-induced cytokine. TWEAK – tumour necrosis factor-related weak inducer of apoptosis. CDCP – complement C1r/C1s. Uegf, Bmp1 (CUB) Domain Containing Protein. FLT3LG – feline McDonough sarcoma (Fms) - related tyrosine kinase 3 ligand. SCF – stem cell factor. uPA – urokinase-type plasminogen activator. CST – cystatin-D. LIFR – leukaemia inhibitory factor receptor. CXCL – C-X-C motif chemokine ligand. HGF – hepatocyte growth factor. TGFA – (pro)transforming growth factor alpha. MMP – matrix metalloproteinase. IFNG – interferon gamma. OSM – oncostatin-M. ENRAGE – extracellular newly identified receptor for advanced glycation end products binding protein.

**Additional file 1: Table S3. *Limma* results for Probiotics and Placebo**

| Proteins | Average NPX (SD) | | logFC | Fold Change | *p* value |
| --- | --- | --- | --- | --- | --- |
|  | Probiotics | Placebo |  |  |  |
| IL-17C | 1.18 (0.55) | 1.49 (0.86) | -0.309 | 0.807 | **0.002** |
| CCL11 | 6.82 (0.54) | 6.96 (0.472 | -0.146 | 0.904 | **0.04** |
| FGF19 | 5.71 (0.92) | 5.96 (1.04) | -0.255 | 0.838 | 0.06 |
| MCP1 | 10.37 (0.41) | 10.47 (0.43) | -0.104 | 0.930 | 0.09 |
| CXCL6 | 8.07 (0.55) | 8.19 (0.47) | -0.119 | 0.921 | 0.10 |
| CDCP1 | 0.68 (0.34) | 0.60 (0.29) | 0.074 | 1.053 | 0.11 |
| TNFRSF9 | 5.80 (0.48) | 5.91 (0.44) | -0.104 | 0.930 | 0.11 |
| CCL23 | 6.52 (0.54) | 6.63 (0.51) | -0.117 | 0.922 | 0.12 |
| CX3CL1 | 1.35 (0.45) | 1.45 (0.56) | -0.104 | 0.930 | 0.15 |
| IL-8 | 4.23 (0.35) | 4.31 (0.48) | -0.086 | 0.942 | 0.15 |
| DNER | 6.83 (0.49) | 6.93 (0.52) | -0.099 | 0.934 | 0.17 |
| CCL20 | 6.16 (0.63) | 6.29 (0.68) | -0.126 | 0.917 | 0.17 |
| HGF | 5.47 (0.38) | 5.54 (0.38) | -0.074 | 0.950 | 0.18 |
| CD40 | 10.40 (0.82) | 10.56 (0.82) | -0.155 | 0.898 | 0.18 |
| CCL3 | 3.91 (0.49) | 4.01 (0.54) | -0.098 | 0.935 | 0.18 |
| TWEAK | 6.86 (0.404) | 6.94 (0.42) | -0.079 | 0.947 | 0.18 |
| OPG | 7.56 (0.48) | 7.65 (0.50) | -0.091 | 0.939 | 0.19 |
| CCL4 | 3. 91 (0.43) | 4.54 (0.56) | -0.091 | 0.939 | 0.20 |
| uPA | 8.17 (0.38) | 8.24 (0.39) | -0.068 | 0.954 | 0.22 |
| CASP8 | 1.46 (0.55) | 1.56 (0.62) | -0.099 | 0.934 | 0.23 |
| LAPTGFB1 | 4.64 (0.404) | 4.71 (0.45) | -0.072 | 0.951 | 0.24 |
| MCP3 | 1.45 (0.56) | 1.54 (0.57) | -0.092 | 0.938 | 0.25 |
| CSF1 | 7.79 (0.59) | 7.88 (0.55) | -0.093 | 0.938 | 0.25 |
| IL-18R1 | 5.94 (0.45) | 6.01 (0.48) | -0.073 | 0.951 | 0.27 |
| AXIN1 | 3.75 (0.80) | 3.86 (0.86) | -0.117 | 0.922 | 0.31 |
| CXCL5 | 10.49 (0.91) | 10.60 (0.73) | -0.117 | 0.922 | 0.31 |
| CD244 | 5.02 (0.53) | 5.10 (0.57) | -0.078 | 0.948 | 0.32 |
| MCP4 | 13.31 (0.63) | 13.39 (0.61) | -0.087 | 0.941 | 0.32 |
| ST1A1 | 3.44 (0.94) | 3.56 (0.97) | -0.123 | 0.919 | 0.36 |
| FLT3LG | 6.49 (0.48) | 6.55 (0.52) | -0.061 | 0.959 | 0.39 |
| CCL25 | 3.81 (0.65) | 3.89 (0.70) | -0.078 | 0.947 | 0.41 |
| PD-L1 | 3.55 (0.41) | 3.59 (0.48) | -0.049 | 0.967 | 0.44 |
| TNFB | 4.45 (0.61) | 4.51 (0.55) | -0.061 | 0.959 | 0.45 |
| IL-10RB | 3.76 (0.47) | 3.81 (0.46) | -0.049 | 0.967 | 0.46 |
| CD6 | 5.47 (0.49) | 5.53 (0.56) | -0.055 | 0.963 | 0.46 |
| ENRAGE | 2.06 (0.46) | 2.12 (0.57) | -0.054 | 0.963 | 0.46 |
| STAMBP | 3.84 (0.71) | 3.92 (0.79) | -0.075 | 0.949 | 0.48 |
| IL-18 | 7.21 (0.73) | 7.15 (0.59) | 0.064 | 1.045 | 0.49 |
| TNF | 1.73 (0.55) | 1.78 (0.55) | -0.050 | 0.966 | 0.53 |
| TNFSF14 | 3.46 (0.48) | 3.49 (0.49) | -0.042 | 0.971 | 0.54 |
| TRAIL | 6.00 (0.43) | 6.04 (0.46) | -0.038 | 0.974 | 0.55 |
| MMP1 | 12.00 (0.86) | 11.93 (0.94) | 0.071 | 1.051 | 0.57 |
| CST5 | 3.73 (0.76) | 3.79 (0.75) | -0.059 | 0.960 | 0.58 |
| ADA | 4.85 (0.58) | 4.89 (0.63) | -0.046 | 0.968 | 0.58 |
| LIFR | 1.85 (0.36) | 1.87 (0.36) | -0.028 | 0.980 | 0.59 |
| CXCL1 | 1.35 (0.45) | 1.45 (0.56) | -0.035 | 0.976 | 0.61 |
| IFNG | 5.73 (1.26) | 5.65 (1.10) | 0.081 | 1.057 | 0.63 |
| SIRT2 | 3.04 (1.05) | 3.11 (1.13) | -0.073 | 0.951 | 0.63 |
| TGFA | 1.54 (0.33) | 1.57 (0.36) | -0.024 | 0.984 | 0.64 |
| IL-12B | 5.44 (0.56) | 5.40 (0.57) | 0.036 | 1.025 | 0.65 |
| CD8A | 7.82 (0.62) | 7.86 (0.65) | -0.040 | 0.973 | 0.66 |
| CD5 | 6.31 (0.68) | 6.35 (0.71) | -0.041 | 0.972 | 0.67 |
| CCL28 | 0.96 (0.35) | 0.94 (0.39) | 0.021 | 1.015 | 0.69 |
| TRANCE | 4.03 (0.54) | 4.06 (0.59) | -0.032 | 0.978 | 0.69 |
| EBP1 | 6.07 (1.12) | 6.10 (1.08) | -0.042 | 0.971 | 0.78 |
| CCL19 | 6.64 (0.61) | 6.66 (0.58) | -0.021 | 0.986 | 0.80 |
| CXCL9 | 5.14 (0.88) | 5.11 (0.77) | 0.027 | 1.019 | 0.81 |
| CXCL11 | 9.30 (0.80) | 9.27 (0.92) | 0.029 | 1.020 | 0.81 |
| CXCL10 | 7.04 (0.94) | 7.07 (0.99) | -0.031 | 0.979 | 0.82 |
| SCF | 6.57 (0.73) | 6.59 (0.66) | -0.021 | 0.985 | 0.83 |
| OSM | 3.23 (0.73) | 3.25 (0.78) | -0.018 | 0.987 | 0.86 |
| VEGFA | 8.78 (0.56) | 8.79 (0.54) | -0.013 | 0.991 | 0.87 |
| MMP10 | 7.67 (0.91) | 7.65 (0.79) | 0.018 | 1.012 | 0.88 |
| MCP2 | 7.07 (0.72) | 7.06 (0.71) | 0.009 | 1.006 | 0.93 |

In bold: p-values <0.05 were considered statistically signficant. NPX – normalised protein expression. SD- standard deviation. *Limma* – Linear Models for Microarray Data. ADA – adenosine deaminase. AXIN – axis inhibitor protein. CASP – caspase. SIRT – sirtuin. ST – sulfotransferase. STAMBP – signal-transducing adaptor molecule-binding protein. CD – cluster of differentiation. PD-L – programmed cell death. TNFSF – tumour necrosis factor ligand superfamily member. 4E-BP – eukaryotic translation initiation factor 4E-binding protein. CCL – C-C motif chemokine ligand. MCP – monocyte chemotactic protein. CSF – macrophage colony-stimulating factor. DNER – delta and notch-like epidermal growth factor-related receptor. FGF – fibroblast growth factor. LAPTGFB1 – latency-associated peptide transforming growth factor beta-1 proprotein. VEGFA – vascular endothelial growth factor A. IL – interleukin. OPG – osteoprotegerin. TNFRSF – tumour necrosis factor receptor superfamily member. TRAIL – tumour necrosis factor-related apoptosis-inducing ligand. TRANCE – tumour necrosis factor-related activation-induced cytokine. TWEAK – tumour necrosis factor-related weak inducer of apoptosis. CDCP – complement C1r/C1s,Uegf, Bmp1 (CUB) Domain Containing Protein. FLT3LG – feline McDonough sarcoma (Fms) - related tyrosine kinase 3 ligand. SCF – stem cell factor. uPA – urokinase-type plasminogen activator. CST – cystatin-D. LIFR – leukaemia inhibitory factor receptor. CXCL – C-X-C motif chemokine ligand. HGF – hepatocyte growth factor. TGFA – (pro)transforming growth factor alpha. MMP – matrix metalloproteinase. IFNG – interferon gamma. OSM – oncostatin-M. ENRAGE – extracellular newly identified receptor for advanced glycation end products binding protein.

**Additional file 1: Table S4. *Limma* results for Cumulative AD and Non-AD in children**

| Proteins | Average NPX (SD) | | logFC | Fold Change | *p* value |
| --- | --- | --- | --- | --- | --- |
|  | AD (n = 49) | Non AD  (n = 153) |  |  |  |
| IL-17C | 1.59 (0.92) | 1.26 (0.65) | 0.331 | 1.258 | **0.005** |
| MCP4 | 13.55 (0.67) | 13.29 (0.59) | 0.257 | 1.195 | **0.01** |
| uPA | 8.32 (0.42) | 8.17 (0.37) | 0.154 | 1.113 | **0.02** |
| CD6 | 5.64 (0.65) | 5.45 (0.47) | 0.194 | 1.144 | **0.02** |
| CASP8 | 1.65 (0.70) | 1.46 (0.54) | 0.185 | 1.137 | 0.05 |
| CST5 | 3.93 (0.84) | 3.70 (0.72) | 0.230 | 1.173 | 0.06 |
| TNFSF14 | 3.58 (0.59) | 3.44 (0.45) | 0.142 | 1.103 | 0.08 |
| SIRT2 | 3.30 (0.99) | 3.00 (1.11) | 0.296 | 1.227 | 0.09 |
| IL-10RB | 3.88 (0.52) | 3.76 (4.40) | 0.122 | 1.088 | 0.11 |
| IL-8 | 4.35 (0.44) | 4.25 (0. 41) | 0.110 | 1.079 | 0.12 |
| AXIN1 | 3.96 (0.75) | 3.75 (0.85) | 0.204 | 1.152 | 0.13 |
| Flt3L | 6.61 (0.52) | 6.49 (0.49) | 0.115 | 1.083 | 0.16 |
| CCL3 | 4.05 (0.58) | 3.93 (0.49) | 0.118 | 1.085 | 0.16 |
| STAMBP | 4.00 (0.73) | 3.84 (0.76) | 0.163 | 1.119 | 0.19 |
| MCP3 | 1.41 (0.58) | 1.53 (0.56) | -0.119 | 0.921 | 0.20 |
| 4E-BP1 | 6.25 (1.28) | 6.03 (1.04) | 0.219 | 1.164 | 0.22 |
| ADA | 4.96 (0.73) | 4.85 (0.55) | 0.113 | 1.081 | 0.25 |
| CD5 | 6.43 (0.82) | 6.30 (0.65) | 0.129 | 1.093 | 0.26 |
| TGFA | 1.60 (0.36) | 1.54 (0.34) | 0.065 | 1.046 | 0.26 |
| CCL4 | 4.57 (0.61) | 4.48 (0.46) | 0.090 | 1.064 | 0.28 |
| CDCP1 | 0.60 (0.30) | 0.65 (0.32) | -0.058 | 0.961 | 0.29 |
| CD8A | 7.92 (0.70) | 7.81 (0.61) | 0.110 | 1.079 | 0.29 |
| SCF | 6.67 (0.73) | 6.55 (0.68) | 0.118 | 1.085 | 0.30 |
| TNFRSF9 | 5.91 (0.54) | 5.83 (0.44) | 0.080 | 1.057 | 0.30 |
| TNFB | 4.40 (0.68) | 4.50 (0.54) | -0.093 | 0.938 | 0.33 |
| CXCL11 | 9.21 (0.74) | 9.31 (0.90) | -0.103 | 0.931 | 0.46 |
| DNER | 6.93 (0.55) | 6.87 (0.50) | 0.060 | 1.043 | 0.47 |
| OPG | 7.65 (0.59) | 7.59 (0.46) | 0.058 | 1.041 | 0.48 |
| CXCL5 | 10.48 (0.92) | 10.57 (0.80) | -0.087 | 0.942 | 0.52 |
| FGF19 | 5.91 (1.07) | 5.81 (0.96) | 0.101 | 1.073 | 0.53 |
| HGF | 5.54 (0.43) | 5.50 (0.37) | 0.040 | 1.028 | 0.53 |
| IL-18R1 | 6.01(0.54) | 5.96 (0.44) | 0.048 | 1.034 | 0.53 |
| TNF | 1.71 (0.47) | 1.76 (0.57) | -0.052 | 0.964 | 0.56 |
| CD244 | 5.10 (0.51) | 5.05 (0.56) | 0.052 | 1.037 | 0.57 |
| IL-18 | 7.23 (0.75) | 7.17 (0.63) | 0.061 | 1.043 | 0.58 |
| MMP1 | 12.03 (0.83) | 11.95 (0.92) | 0.082 | 1.058 | 0.58 |
| CCL20 | 6.27 (0.74) | 6.21 (0.63) | 0.059 | 1.042 | 0.59 |
| IL-12B | 5.38 (0.56) | 5.43 (0.57) | -0.049 | 0.967 | 0.60 |
| MCP1 | 10.44 (0.46) | 10.41(0.42) | 0.036 | 1.025 | 0.61 |
| CX3CL1 | 1.43 (0.67) | 1.39 (0.45) | 0.041 | 1.029 | 0.63 |
| CXCL10 | 7.11 (0.87) | 7.04 (0.99) | 0.073 | 1.052 | 0.64 |
| CCL25 | 3.82 (0.67) | 3.87 (0.68) | -0.050 | 0.966 | 0.65 |
| IFNG | 5.76 (1.03) | 5.67 (1.23) | 0.086 | 1.061 | 0.66 |
| TWEAK | 6.92 (0.46) | 6.89 (0.40) | 0.029 | 1.020 | 0.67 |
| CSF1 | 7.86 (0.66) | 7.83 (0.55) | 0.037 | 1.026 | 0.70 |
| LIFR | 1.88 (0.42) | 1.85 (0.34) | 0.022 | 1.016 | 0.71 |
| TRAIL | 6.00 (0.50) | 6.03 (0.42) | -0.027 | 0.982 | 0.72 |
| TRANCE | 4.07 (0.60) | 4.04 (0.56) | 0.033 | 1.023 | 0.73 |
| VEGFA | 8.81(0.58) | 8.78 (0.54) | 0.030 | 1.021 | 0.74 |
| CCL11 | 6.91(0.54) | 6.88 (0.50) | 0.027 | 1.019 | 0.74 |
| MMP10 | 7.69 (0.92) | 7.65 (0.83) | 0.043 | 1.030 | 0.76 |
| LAPTGFB1 | 4.69 (0.38) | 4.67 (0.45) | 0.022 | 1.015 | 0.76 |
| CD40 | 10.51(0.77) | 10.47 (0.84) | 0.042 | 1.029 | 0.76 |
| CXCL6 | 8.11 (0.50) | 8.14 (0.52) | -0.025 | 0.983 | 0.78 |
| CCL23 | 6.59 (0.57) | 6.57 (0.51) | 0.024 | 1.017 | 0.78 |
| ENRAGE | 2.07 (0.53) | 2.10 (0.52) | -0.023 | 0.984 | 0.79 |
| OSM | 3.26 (0.80) | 3.23 (0.74) | 0.026 | 1.018 | 0.84 |
| MCP2 | 7.08 (0.64) | 7.06 (0.74) | 0.019 | 1.013 | 0.87 |
| CXCL1 | 9.30 (0.46) | 9.29 (0.49) | 0.012 | 1.009 | 0.88 |
| CXCL9 | 5.11 (0.79) | 5.13 (0.84) | -0.019 | 0.987 | 0.89 |
| CCL19 | 6.65 (0.57) | 6.64 (0.60) | 0.009 | 1.006 | 0.92 |
| PD-L1 | 3.57 (0.37) | 3.57 (0.47) | -0.002 | 0.999 | 0.98 |
| ST1A1 | 3.50 (0.92) | 3.50 (0.97) | -0.003 | 0.998 | 0.99 |
| CCL28 | 0.95 (0.36) | 0.95 (0.37) | -0.001 | 0.999 | 0.99 |

In bold: p-values <0.05 were considered statistically signficant. NPX – normalised protein expression. SD- standard deviation. *Limma* – Linear Models for Microarray Data. ADA – adenosine deaminase. AXIN – axis inhibitor protein. CASP – caspase. SIRT – sirtuin. ST – sulfotransferase. STAMBP – signal-transducing adaptor molecule-binding protein. CD – cluster of differentiation. PD-L – programmed cell death. TNFSF – tumour necrosis factor ligand superfamily member. 4E-BP – eukaryotic translation initiation factor 4E-binding protein. CCL – C-C motif chemokine ligand. MCP – monocyte chemotactic protein. CSF – macrophage colony-stimulating factor. DNER – delta and notch-like epidermal growth factor-related receptor. FGF – fibroblast growth factor. LAPTGFB1 – latency-associated peptide transforming growth factor beta-1 proprotein. VEGFA – vascular endothelial growth factor A. IL – interleukin. OPG – osteoprotegerin. TNFRSF – tumour necrosis factor receptor superfamily member. TRAIL – tumour necrosis factor-related apoptosis-inducing ligand. TRANCE – tumour necrosis factor-related activation-induced cytokine. TWEAK – tumour necrosis factor-related weak inducer of apoptosis. CDCP – complement C1r/C1s, Uegf, Bmp1 (CUB) Domain Containing Protein. FLT3LG – feline McDonough sarcoma (Fms) - related tyrosine kinase 3 ligand. SCF – stem cell factor. uPA – urokinase-type plasminogen activator. CST – cystatin-D. LIFR – leukaemia inhibitory factor receptor. CXCL – C-X-C motif chemokine ligand. HGF – hepatocyte growth factor. TGFA – (pro)transforming growth factor alpha. MMP – matrix metalloproteinase. IFNG – interferon gamma. OSM – oncostatin-M. ENRAGE – extracellular newly identified receptor for advanced glycation end products binding protein.

**Additional file 1: Table S5: Expressions of proteins in first group by sample clusters**

|  | Mean NPX (SD) | | | | Mean difference (95 % CI) | | | |
| --- | --- | --- | --- | --- | --- | --- | --- | --- |
| Protein | **Cluster 1** | **Cluster 2** | **Cluster 3** | **Cluster 4** | **Cluster 2 vs 1** | **Cluster 3 vs 1** | **Cluster 4 vs 1** |  |
| TNFSF14 | 3.53 (0.55) | 3.58 (0.41) | 3.29 (0.37) | 3.88 (0.73) | 0.05  (-0.12, 0.22) | **-0.23**  **(-0.39, -0.07)** | **0.35**  **(0.06, 0.65)** |  |
| CD6 | 5.59 (0.47) | 5.67 (0.56) | 5.32 (0.48) | 5.37 (0.59) | 0.08  (-0.11, 0.26) | **-0.27**  **(-0.45, -0.09)** | **-0.22**  **(-0.54, 0.10)** |  |
| CD8A | 7.96 (0.63) | 8.11 (0.59) | 7.65 (0.52) | 7.12 (0.67) | 0.15  (-0.06, 0.37) | **-0.31**  **(-0.51, -0.11)** | **-0.84**  **(-1.20, -0.48)** |  |
| CD5 | 6.46 (0.69) | 6.18 (0.65) | 6.24 (0.61) | 7.04 (0.96) | **-0.28**  **(-0.53, -0.04)** | -0.23  (-0.46, 0.01) | **0.58**  **(0.16, 1.00)** |  |
| CASP8 | 1.88 (0.48) | 1.48 (0.38) | 1.41 (0.39) | 1.97 (0.43) | **-0.40**  **(-0.55, -0.25)** | **-0.47**  **(-0.62, -0.33)** | 0.09  (-0.17, 0.35) |  |
| CD40 | 11.11 (0.75) | 10.01 (0.51) | 10.31 (0.77) | 10.92 (0.83) | **-1.09**  **(-1.35, -0.84)** | **-0.80**  **(-1.04, -0.56)** | -0.19  (-0.63, 0.25) |  |
| ADA | 5.14 (0.67) | 4.62 (0.29) | 4.73 (0.53) | 5.81 (0.49) | **-0.52**  **(-0.71, -0.33)** | **-0.41**  **(-0.59, -0.23)** | **0.67**  **(0.35, 1.00)** |  |
| 4E-BP1 | 6.55 (0.92) | 5.77 (0.90) | 5.71 (1.00) | 7.79 (1.01) | **-0.78**  **(-1.13, -0.43)** | **-0.85**  **(-1.18, -0.51)** | **1.24**  **(0.64, 1.83)** |  |
| ST1A1 | 4.22 (0.62) | 3.00 (0.79) | 3.29 (0.95) | 3.86 (0.99) | **-1.22**  **(-1.53, -0.92)** | **-0.93**  **(-1.22, -0.64)** | -0.36  (-0.88, 0.15) |  |
| PD-L1 | 3.85 (0.48) | 3.60 (0.29) | 3.36 (0.37) | 3.46 (0.60) | **-0.25**  **(-0.40 , -0.10)** | **-0.50**  **(-0.64, -0.36)** | **-0.39**  **(-0.64, -0.14)** |  |
| CD244 | 5.48 (0.50) | 4.86 (0.44) | 4.92 (0.46) | 5.02 (0.75) | **-0.62**  **(-0.80 , -0.44)** | **-0.56**  **(-0.73, -0.39)** | **-0.45**  **(-0.76, -0.15)** |  |
| AXIN1 | 4.60 (0.52) | 3.53 (0.79) | 3.48 (0.65) | 3.47 (0.82) | **-1.07**  **(-1.32 , -0.82)** | **-1.11**  **(-1.35, -0.88)** | **-1.13**  **(-1.55, -0.70)** |  |
| SIRT2 | 4.07 (0.57) | 2.92 (0.78) | 2.88 (0.63) | 3.40 (0.46) | **-1.15**  **(-1.39 , -0.91)** | **-1.19**  **(-1.42, -0.97)** | **-0.67**  **(-1.08, -0.26)** |  |
| STAMBP | 4.64 (0.50) | 3.47 (0.64) | 3.61 (0.60) | 4.09 (0.39) | **-1.17**  **(-1.38 , -0.96)** | **-1.03**  **(-1.23, -0.83)** | **-0.55**  **(-0.91, -0.19)** |  |

In bold: p-values <0.05 were considered statistically signficant. NPX – normalised protein expression. SD- standard deviation. CI – confidence interval. ADA – adenosine deaminase. AXIN – axis inhibitor protein. CASP – caspase. SIRT – sirtuin. STAMBP – signal-transducing adaptor molecule-binding protein. CD – cluster of differentiation. PD-L – programmed cell death. TNFSF – tumour necrosis factor ligand superfamily member. 4E-BP – eukaryotic translation initiation factor 4E-binding protein.

**Additional file 1: Table S6: Expressions of proteins in second group by sample clusters**

|  | **Mean NPX (SD)** | | | | **Mean difference (95 % CI)** | | |
| --- | --- | --- | --- | --- | --- | --- | --- |
| **Protein** | **Cluster 1** | **Cluster 2** | **Cluster 3** | **Cluster 4** | **Cluster 2 vs 1** | **Cluster 3 vs 1** | **Cluster 4 vs 1** |
| TRANCE | 4.21 (0.43) | 4.27 (0.51) | 3.90 (0.51) | 3.04 (0.40) | 0.06  (-0.12, 0.24) | **-0.31**  **(-0.48, -0.14)** | **-1.17**  **(-1.47, -0.87)** |
| CST5 | 3.96 (0.67) | 4.02 (0.57) | 3.67 (0.63) | 2.12 (0.47) | 0.06  (-0.16, 0.29) | **-0.29**  **(-0.51, -0.08)** | **-1.84**  **(-2.23, -1.45)** |
| LAP-TGFB1 | 4.79 (0.30) | 4.88 (0.37) | 4.54 (0.37) | 3.95 (0.58) | 0.09  (-0.04, 0.23) | **-0.25**  **(-0.38, -0.12)** | **-0.84**  **(-1.07, -0.61)** |
| MCP1 | 10.49 (0.37) | 10.59 (0.39) | 10.35 (0.36) | 9.67 (0.40) | 0.10  (-0.04, 0.23) | -0.14  (-0.27, -0.01) | **-0.83**  **(-1.06, -0.59)** |
| uPA | 8.31 (0.27) | 8.41 (0.29) | 8.11 (0.30) | 7.32 (0.31) | **0.10**  **(-0.00, 0.21)** | **-0.20**  **(-0.30, -0.10)** | **-0.99**  **(-1.17, -0.81)** |
| TNFB | 4.60 (0.50) | 4.74 (0.53) | 4.35 (0.45) | 3.40 (0.44) | 0.14  (-0.04, 0.32) | **-0.26**  **(-0.42, -0.09)** | **-1.20**  **(-1.50, -0.89)** |
| CDCP1 | 0.71 (0.32) | 0.86 (0.21) | 0.61 (0.25) | 0.35 (0.26) | **0.15**  **(0.05, 0.24)** | **-0.10**  **(-0.19, -0.01)** | **-0.35**  **(-0.52, -0.19)** |
| CX3CL1 | 1.51 (0.52) | 1.67 (0.34) | 1.30 (0.33) | 0.74 (0.44) | 0.16  (0.01, 0.31) | -0.20  (-0.34, -0.06) | **-0.77**  **(-1.02, -0.51)** |
| TWEAK | 6.97 (0.28) | 7.14 (0.32) | 6.82 (0.30) | 5.87 (0.20) | **0.18**  **(0.07, 0.29)** | **-0.15**  **(-0.25, -0.05)** | **-1.10**  **(-1.28, -0.91)** |
| SCF | 6.65 (0.53) | 6.83 (0.62) | 6.53 (0.66) | 5.28 (0.47) | 0.18  (-0.04, 0.40) | -0.13  (-0.34, 0.09) | **-1.37**  **(-1.75, -0.99)** |
| LIFR | 1.89 (0.20) | 2.10 (0.24) | 1.79 (0.27) | 1.14 (0.20) | **0.21**  **(0.12, 0.30)** | **-0.10**  **(-0.18, -0.02)** | **-0.75**  **(-0.91, -0.60)** |
| MCP3 | 1.68 (0.29) | 1.89 (0.32) | 1.64 (0.29) | 1.58 (0.27) | **0.21**  **(0.10, 0.32)** | -0.04  (-0.14, 0.06) | -0.10  (-0.29, 0.08) |
| IL-12B | 5.48 (0.44) | 5.70 (0.53) | 5.35 (0.40) | 4.19 (0.41) | **0.22**  **(0.06, 0.39)** | -0.13  (-0.28, 0.03) | **-1.29**  **(-1.58, -1.01)** |
| IL-18 | 7.30 (0.67) | 7.54 (0.61) | 6.95 (0.47) | 6.26 (0.57) | **0.24**  **(0.03 , 0.45)** | **-0.35**  **(-0.55 , -0.15)** | **-1.04**  **(-1.40 , -0.68)** |
| VEGFA | 8.86 (0.45) | 9.11 (0.46) | 8.65 (0.48) | 7.80 (0.26) | **0.25**  **(0.08, 0.41)** | **-0.21**  **(-0.37, -0.05)** | **-1.06**  **(-1.35, -0.77)** |
| CCL11 | 6.97 (0.39) | 7.22 (0.39) | 6.73 (0.37) | 5.86 (0.50) | **0.25**  **(0.11, 0.40)** | **-0.24**  **(-0.38, -0.10)** | **-1.11**  **(-1.36, -0.87)** |
| IL-18R1 | 6.04 (0.41) | 6.30 (0.38) | 5.79 (0.33) | 5.19 (0.40) | **0.26**  **(0.12, 0.40)** | **-0.25**  **(-0.38, -0.12)** | **-0.85**  **(-1.08, -0.62)** |
| MCP4 | 13.44 (0.57) | 13.70 (0.56) | 13.16 (0.50) | 12.45 (0.47) | **0.26**  **(0.06, 0.46)** | **-0.28**  **(-0.47, -0.09)** | **-0.99**  **(-1.33, -0.65)** |
| Flt3L | 6.58 (0.43) | 6.85 (0.37) | 6.37 (0.39) | 5.58 (0.41) | **0.27**  **(0.13, 0.42)** | **-0.21**  **(-0.35, -0.07)** | **-1.00**  **(-1.25, -0.75)** |
| CCL23 | 6.65 (0.38) | 6.93 (0.38) | 6.43 (0.39) | 5.40 (0.39) | **0.28**  **(0.14, 0.42)** | **-0.23**  **(-0.36, -0.09)** | **-1.26**  **(-1.50, -1.01)** |
| CCL19 | 6.75 (0.50) | 7.03 (0.42) | 6.43 (0.53) | 5.62 (0.25) | **0.29**  **(0.11, 0.46)** | **-0.32**  **(-0.48, -0.15)** | **-1.12**  **(-1.43, -0.82)** |
| IL-10RB | 3.83 (0.31) | 4.12 (0.33) | 3.66 (0.36) | 2.73 (0.30) | **0.30**  **(0.17, 0.42)** | **-0.16**  **(-0.28, -0.05)** | **-1.09**  **(-1.30, -0.88)** |
| DNER | 6.91 (0.34) | 7.21 (0.37) | 6.80 (0.40) | 5.65 (0.35) | **0.30**  **(0.16, 0.43)** | -0.11  (-0.24, 0.02) | **-1.27**  **(-1.50, -1.04)** |
| FGF19 | 6.13 (0.74) | 6.45 (0.97) | 5.32 (0.78) | 4.68 (0.61) | **0.31**  **(0.01, 0.62)** | **-0.82**  **(-1.10, -0.53)** | **-1.45**  **(-1.97, -0.94)** |
| TRAIL | 6.06 (0.31) | 6.38 (0.31) | 5.88 (0.29) | 4.98 (0.18) | **0.32**  **(0.21, 0.43)** | **-0.18**  **(-0.29, -0.08)** | **-1.08**  **(-1.26, -0.89)** |
| TNFRSF9 | 5.88 (0.31) | 6.20 (0.35) | 5.73 (0.33) | 4.78 (0.39) | **0.32**  **(0.20, 0.45)** | **-0.15**  **(-0.26, -0.03)** | **-1.10**  **(-1.31, -0.89)** |
| OPG | 7.64 (0.31) | 7.97 (0.29) | 7.49 (0.38) | 6.41 (0.41) | **0.33**  **(0.20, 0.45)** | **-0.15**  **(-0.27, -0.03)** | **-1.24**  **(-1.45, -1.02)** |
| CCL25 | 4.00 (0.54) | 4.34 (0.62) | 3.52 (0.39) | 2.87 (0.82) | **0.34**  **(0.14, 0.53)** | **-0.48**  **(-0.66, -0.29)** | **-1.13**  **(-1.47, -0.80)** |
| TNF | 1.74 (0.41) | 2.11 (0.52) | 1.61 (0.48) | 0.91 (0.30) | **0.37**  **(0.20, 0.54)** | -0.13  (-0.30, 0.03) | **-0.83**  **(-1.13, -0.54)** |
| CCL20 | 6.30 (0.57) | 6.74 (0.62) | 5.88 (0.40) | 5.41 (0.43) | **0.44**  **(0.25, 0.64)** | **-0.42**  **(-0.60, -0.23)** | **-0.89**  **(-1.22, -0.57)** |
| CSF1 | 7.84  (0.38) | 8.37  (0.29) | 7.64 (0.35) | 6.44 (0.40) | **0.53**  **(0.40, 0.66)** | **-0.20**  **(-0.32, -0.07)** | **-1.39**  **(-1.61, -1.18)** |

In bold: p-values <0.05 were considered statistically signficant. NPX – normalised protein expression. SD- standard deviation. CI – confidence interval. CCL – C-C motif chemokine ligand. MCP – monocyte chemotactic protein. CSF – macrophage colony-stimulating factor. DNER – delta and notch-like epidermal growth factor-related receptor. FGF – fibroblast growth factor. LAPTGFB1 – latency-associated peptide transforming growth factor beta-1 proprotein. VEGFA – vascular endothelial growth factor A. IL – interleukin. OPG – osteoprotegerin. TNFRSF – tumour necrosis factor receptor superfamily member. TRAIL – tumour necrosis factor-related apoptosis-inducing ligand. TRANCE – tumour necrosis factor-related activation-induced cytokine. TWEAK – tumour necrosis factor-related weak inducer of apoptosis. CDCP – complement C1r/C1s, Uegf, Bmp1 (CUB) Domain Containing Protein. FLT3LG – feline McDonough sarcoma (Fms) - related tyrosine kinase 3 ligand. SCF – stem cell factor. uPA – urokinase-type plasminogen activator. CST – cystatin-D. LIFR – leukaemia inhibitory factor receptor.

**Additional file 1: Table S7: Expressions of proteins in third group by sample clusters**

|  | **Mean NPX (SD)** | | | | **Mean difference (95 % CI)** | | |
| --- | --- | --- | --- | --- | --- | --- | --- |
| **Protein** | **Cluster 1** | **Cluster 2** | **Cluster 3** | **Cluster 4** | **Cluster 2 vs 1** | **Cluster 3 vs 1** | **Cluster 4 vs 1** |
| CXCL1 | 9.57 (0.39) | 9.33 (0.51) | 9.11 (0.42) | 8.89 (0.40) | **-0.24**  **(-0.40, -0.08)** | **-0.46**  **(-0.61, -0.31)** | **-0.68**  **(-0.96, -0.41)** |
| CCL28 | 1.18 (0.24) | 1.13 (0.20) | 1.07 (0.19) | 0.99 (0.20) | -0.05  (-0.13, 0.03) | **-0.11**  **(-0.18, -0.04)** | **-0.19**  **(-0.32, -0.06)** |
| CXCL5 | 10.78 (0.60) | 10.80 (0.81) | 10.33 (0.84) | 9.57 (0.80) | 0.02  (-0.26, 0.31) | **-0.45**  **(-0.72, -0.18)** | **-1.21**  **(-1.69, -0.73)** |
| CXCL6 | 8.29 (0.39) | 8.35 (0.51) | 7.95 (0.47) | 7.53 (0.37) | 0.06  (-0.11, 0.23) | **-0.34**  **(-0.50, -0.18)** | **-0.76**  **(-1.04, -0.47)** |
| CCL4 | 4.64 (0.53) | 4.71 (0.38) | 4.32 (0.46) | 3.92 (0.24) | 0.07  (-0.10, 0.23) | **-0.32**  **(-0.48, -0.17)** | **-0.72**  **(-1.00, -0.44)** |
| CCL3 | 4.11 (0.56) | 4.19 (0.35) | 3.72 (0.43) | 3.62 (0.66) | 0.08  (-0.09, 0.25) | **-0.39**  **(-0.56, -0.23)** | **-0.49**  **(-0.78, -0.20)** |
| IL-8 | 4.28 (0.33) | 4.46 (0.53) | 4.16 (0.32) | 3.99 (0.40) | **0.19**  **(0.04, 0.33)** | -0.12  (-0.26, 0.02) | **-0.29**  **(-0.54, -0.04)** |
| IFNG | 5.97 (1.31) | 6.18 (1.08) | 5.27 (0.88) | 4.75 (0.90) | 0.21  (-0.19, 0.60) | **-0.70**  **(-1.08, -0.33)** | **-1.23**  **(-1.90, -0.55)** |
| MCP2 | 7.17 (0.80) | 7.42 (0.71) | 6.86 (0.44) | 6.15 (0.47) | **0.25**  **(0.01, 0.48)** | **-0.32**  **(-0.54, -0.10)** | **-1.02**  **(-1.42, -0.62)** |
| MMP1 | 12.07 (0.71) | 12.34 (1.00) | 11.65 (0.85) | 11.60 (0.74) | 0.27  (-0.04, 0.59) | **-0.41**  **(-0.71, -0.12)** | -0.46  (-1.00, 0.07) |
| HGF | 5.49 (0.26) | 5.83 (0.28) | 5.36 (0.30) | 4.90 (0.49) | **0.34**  **(0.23, 0.45)** | **-0.13**  **(-0.23, -0.03)** | **-0.60**  **(-0.78, -0.41)** |
| CXCL11 | 9.37 (0.91) | 9.75 (0.85) | 8.92 (0.61) | 8.93 (0.96) | **0.38**  **(0.09, 0.68)** | **-0.45**  **(-0.72, -0.17)** | -0.43  (-0.93 , 0.06) |
| CXCL10 | 7.25 (1.06) | 7.63 (0.90) | 6.64 (0.60) | 5.96 (0.46) | **0.39**  **(0.08, 0.69)** | **-0.61**  **(-0.90, -0.32)** | **-1.28**  **(-1.81, -0.76)** |
| TGFA | 1.46 (0.28) | 1.85 (0.33) | 1.43 (0.23) | 1.33 (0.42) | **0.40**  **(0.29, 0.50)** | -0.03  (-0.13, 0.07) | -0.13  (-0.31, 0.05) |
| CXCL9 | 5.22 (0.84) | 5.67 (0.76) | 4.73 (0.52) | 4.53 (0.96) | **0.46**  **(0.19, 0.72)** | **-0.49**  **(-0.74, -0.24)** | **-0.69**  **(-1.14, -0.24)** |
| IL-17C | 1.44 (0.42) | 1.90 (0.74) | 1.30 (0.51) | 0.88 (0.35) | **0.46**  **(0.25, 0.66)** | -0.14  (-0.34, 0.05) | **-0.56**  **(-0.91, -0.21)** |
| EN-RAGE | 1.94 (0.40) | 2.48 (0.56) | 1.92 (0.36) | 2.02 (0.68) | **0.54**  **(0.37, 0.71)** | -0.02  (-0.18, 0.14) | 0.08  (-0.21, 0.36) |
| MMP10 | 7.46 (0.63) | 8.17 (0.72) | 7.62 (0.82) | 6.33 (0.62) | **0.71**  **(0.44, 0.98)** | 0.16  (-0.09, 0.42) | **-1.12**  **(-1.58, -0.66)** |
| OSM | 2.92 (0.59) | 3.74 (0.79) | 3.05 (0.62) | 3.42 (0.78) | **0.82**  **(0.57, 1.07)** | 0.13  (-0.11, 0.37) | 0.49  (0.07, 0.92) |

In bold: p-values <0.05 were considered statistically signficant. . NPX – normalised protein expression. SD- standard deviation. CI – confidence interval. CCL – C-C motif chemokine ligand. MCP – monocyte chemotactic protein. IL – interleukin. CXCL – C-X-C motif chemokine ligand. TGFA – (pro)transforming growth factor alpha. MMP – matrix metalloproteinase. IFNG – interferon gamma. OSM – oncostatin-M. ENRAGE – extracellular newly identified receptor for advanced glycation end products binding protein.

**Additional figure legends**

**Additional file 1:** Figure S1. Inflammatory proteins of 2-year-old children following maternal probiotics suplementation
